# Optimizing Vaccine Allocation at Different Points in Time during an Epidemic

**DOI:** 10.1371/journal.pone.0013767

**Published:** 2010-11-11

**Authors:** Laura Matrajt, Ira M. Longini

**Affiliations:** 1 Department of Applied Mathematics, University of Washington, Seattle, Washington, United States of America; 2 Center for Statistics and Quantitative Infectious Diseases, Vaccine and Infectious Disease Division, Fred Hutchinson Cancer Research Center, Seattle, Washington, United States of America; 3 Department of Biostatistics, School of Public Health, University of Washington, Seattle, Washington, United States of America; The University of Hong Kong, Hong Kong

## Abstract

**Background:**

Pandemic influenza A(H1N1) 2009 began spreading around the globe in April of 2009 and vaccination started in October of 2009. In most countries, by the time vaccination started, the second wave of pandemic H1N1 2009 was already under way. With limited supplies of vaccine, we are left to question whether it may be a good strategy to vaccinate the high-transmission groups earlier in the epidemic, but it might be a better use of resources to protect instead the high-risk groups later in the epidemic. To answer this question, we develop a deterministic epidemic model with two age-groups (children and adults) and further subdivide each age group in low and high risk.

**Methods and Findings:**

We compare optimal vaccination strategies started at various points in time in two different settings: a population in a developed country where children account for 24% of the population, and a population in a less developed country where children make up the majority of the population, 55%. For each of these populations, we minimize mortality or hospitalizations and we find an optimal vaccination strategy that gives the best vaccine allocation given a starting vaccination time and vaccine coverage level. We find that population structure is an important factor in determining the optimal vaccine distribution. Moreover, the optimal policy is dynamic as there is a switch in the optimal vaccination strategy at some time point just before the peak of the epidemic. For instance, with 25% vaccine coverage, it is better to protect the high-transmission groups before this point, but it is optimal to protect the most vulnerable groups afterward.

**Conclusions:**

Choosing the optimal strategy before or early in the epidemic makes an important difference in minimizing the number of influenza infections, and consequently the number of influenza deaths or hospitalizations, but the optimal strategy makes little difference after the peak.

## Introduction

For the pandemic H1N1 2009 influenza, vaccine production started in the early summer of 2009. Several countries immediately ordered vaccine [1], [2], with the hope that the first production batches would be ready in the early fall of 2009. This was not the case, however, and for most countries vaccine arrived much later than predicted. Meanwhile, the World Health Organization (WHO) expected to supply 95 low-and middle-income countries with enough vaccine to cover 10% of their populations [3]. When vaccine supplies are limited, vaccinating the high-transmission groups, such as school children or young adults, has proven to be a good strategy for preventing the spread of the disease, and by doing so the groups at high risk will be indirectly protected [4], [5], [6], [7]. While this strategy makes sense earlier in the epidemic, this might not be the optimal use of vaccine once the epidemic has begun. Indeed, once there is a large proportion of the high-transmission groups infected and later on immune, vaccine would probably have little effect in these groups and could be more effectively used in the high-risk groups, giving them direct protection. When and who should receive vaccine first is still an open question. Recent advances have been made in this direction [8], [9], [10], [11], [12], but the problem is complex and depends on multiple factors. For instance, the optimal use of vaccines depends on the population structure: countries or cities where school children or college students make up large proportions of the population will have different epidemic dynamics than a country where these younger people make up a smaller proportion of the population. Therefore, different countries with different socioeconomic backgrounds will have different epidemic dynamics, and should consequently optimize their resources according to their needs.

In the present work, we developed a deterministic model with two groups, children and adults, and we further divided each of these age groups into low and high risk. We compared optimal vaccination strategies in two different settings: 1. A population in a developed country (in what follows denoted by DC), where the children make up 24% of the population [13], and 2. a population in a less developed country (in what follows denoted by LDC), where the children account for 55% of the population [14]. Examples of countries with the former population structure would be countries like the United States, France, United Kingdom or Australia, while examples of countries with the latter structure would include Senegal, Cameroon, and Bolivia [14]. For each of these populations, we minimize mortality or hospitalizations, and we find an optimal vaccination strategy that gives us the best vaccine allocation given a starting vaccination time and supply of vaccine.

## Methods

### Model Assumptions

Our model for influenza is based on the 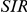 model (see Text S1 for the detailed model). We considered a closed population of size 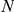. Since influenza has a very short time scale compared to immigration or demographics, none of these features are included. We divided the population into two sub-populations of children and adults of size 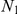 and 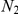, so that 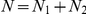. Furthermore, within each sub-population, we divide members into high risk and low risk. Members in each group are either susceptible, infected asymptomatic, infected symptomatic or recovered and immune. In addition, people can be either vaccinated or unvaccinated. The following assumptions were made.

- A fraction 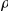 of the infected people will never develop symptoms but will still transmit the infection to others. Asymptomatic infected people have their infectiousness reduced by a factor 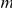 compared to symptomatic infected people, where 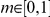.
- Let 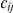 be the number of contacts per day between people in age group 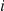 and people in age group 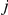, where 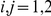. We assumed 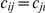.

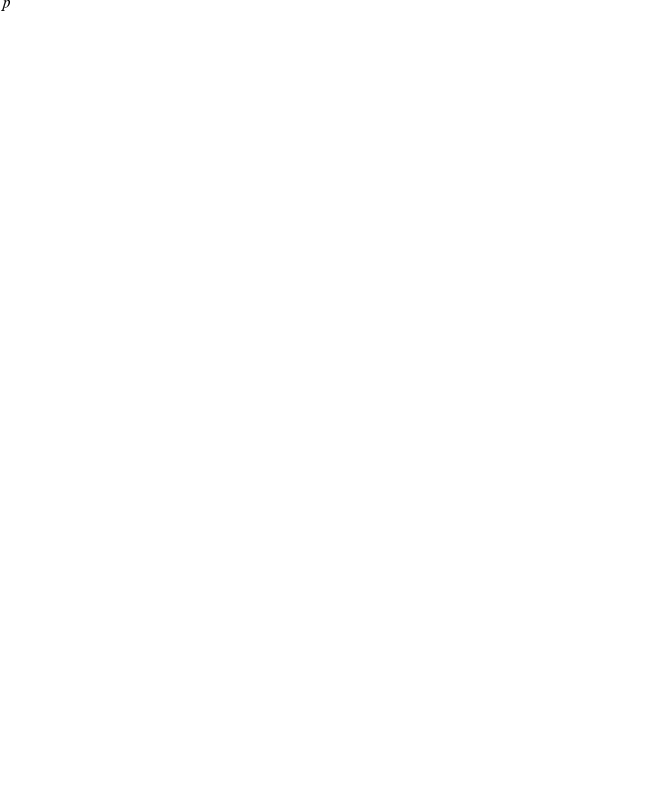
 is the probability of infection given contact.
- People are infectious as soon as they get infected, and they will stay infectious for an average of 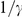 time units, where 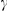 is the recovery rate.
- Following the ideas in [15], vaccination has three major effects:

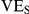
, the vaccine efficacy for susceptibility, which is the ability of the vaccine to prevent infection.

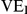
, the vaccine efficacy for infectiousness, which is the effect of the vaccine in reducing infectiousness and transmission to others.

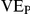
, the vaccine efficacy for pathogenicity, which accounts for the effect of the vaccine in reducing the symptoms given infection.
- The effect of each of the efficacies builds monotonically in time according to expontial-like functions. Based on previous immunogenicity studies (e.g., [16], [17], [18], [19]), we assumed that the vaccine efficacy will reach its full potential 14 days after being administered. In addition, we assumed that the vaccine efficacies do not wane over the time period being modeled.

A detailed description of the model can be found in Text S1.

### Model calibration

We used the United States (US) as a basis for the developed country setting. We then calibrated this model for the pandemic H1N1 2009 in the US according to the numbers given in table S2 to obtain the final illness attack rates (defined as the percentage of the population that became ill) shown in table S3. For the less developed country setting, the parameters were taken to be all identical to those from the US except for the proportion of children in the population, the influenza-related mortality, and the influenza-related hospitalizations. Since this kind of data is rarely collected and difficult to obtain for less developed countries, we assumed two extreme scenarios. First, we assumed that both the rates of influenza-related mortality and hospitalizations for each group in a less developed country were exactly the same as the ones in the US. Then, we used infant mortality rate in less developed countries as a proxy for the excess of influenza-related mortality and hospitalizations in children in less developed countries. The infant mortality rate was computed as an average of five less developed countries among the ones receiving vaccine from WHO, based on the numbers given in [14]. That is, we computed the ratio of the average infant mortality rate in less developed countries and the infant mortality rate in the US and adjusted the influenza-related mortality rate and the hospitalization rate in less developed countries by multiplying them by this factor. Similarly, based on [20] we used excess in female adult mortality as a proxy for the influenza-related mortality and hospitalizations in the adult group. Here, we used hospitalizations in a LDC as a proxy for severity. The number of hospitalizations represents the number of severe cases that would require medical attention, but not necessarily the number of cases that will go to the hospital. This is because in a LDC, the health seeking behavior and hospital capacity might be extremely different from the DC setting, and extrapolation might not be adequate.

### Implementing vaccination

**Figure 1:**
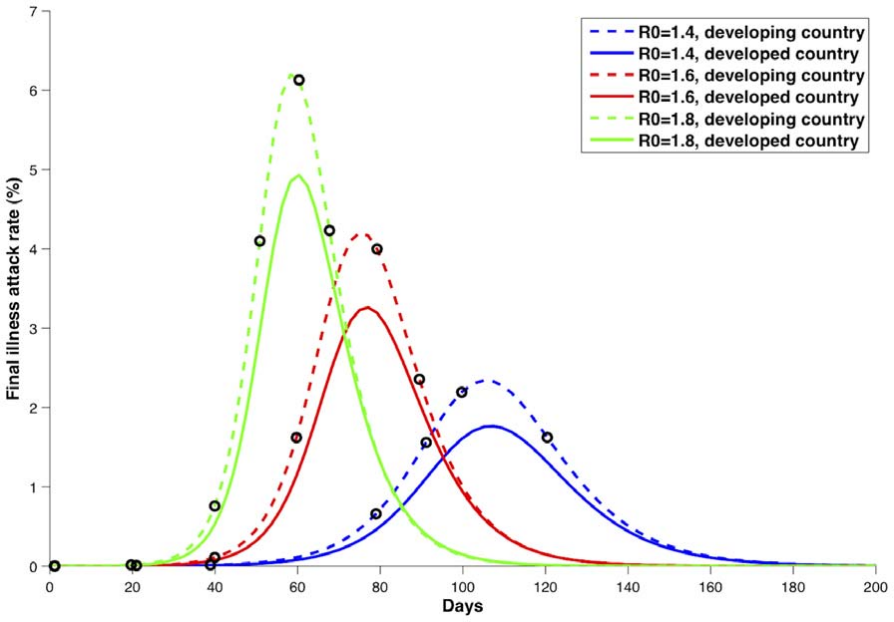
Epidemic curves for both the less developed country (LDC) and the developed country (DC). **Caption:** Epidemic curves for all the basic reproduction numbers considered and for both the less developed country (LDC) and the developed country (DC) in absence of vaccination. The circles in black denote the starting vaccination times considered for each given 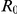.

Based on current estimates [21], [22], [23], we considered the basic reproduction number 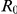 to be in the set 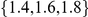. The basic reproduction numbers were computed following the approach given in [24] and [25], [26]. We investigated the influence of the timing of the vaccination program on its effect. To do this, we considered, for each basic reproduction number, six different times for starting vaccination: two of them before the exponential phase of the epidemic, two of them during the exponential phase of the epidemic, one close the peak of the epidemic curve, and one further after the peak (figure 1 and table S1).

The World Health Organization (WHO) has pledged to give enough vaccine to less developed countries to cover 10% of their populations [27]. At the same time, vaccine in developed countries was delivered in batches. In the US, the first batch of vaccine was assumed to be enough for 20% of the population [28]. With this in mind, we considered vaccine supplies with enough to cover 2%, 15% or 25% of the population.

For simplicity, we assumed for a given coverage that all the vaccine is delivered at once, however, vaccinated people acquire their protection gradually as the vaccine efficacies build up over time (figure S1). For further details, the kinetics of the vaccine effects are given in Text S1.

### Optimization

Define a vaccination control vector 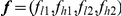. For each possible vector 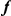, 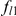 and 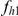 are the fractions of vaccinated children at low and high risk, respectively, and 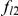 and 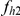 are the fractions of vaccinated adults at low and high risk, respectively. Using a line search optimization algorithm in MATLAB, we determined the vector 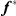 that would give us the optimal vaccine distribution for minimizing either the total number of deaths or the total number of hospitalizations for each of the vaccination initialization times and vaccine coverages given above. Thus, the vector 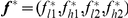 gives us the fractions 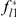 and 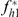 of children at low and high risk, and the fractions 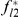 and 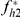 of adults at low and high risk respectively that would minimize total mortality or total hospitalizations during the entire epidemic. Further details are given in Text S1.

## Results

For this analysis, we will focus on a basic reproduction number of 1.6. The results for 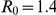 and 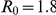 are summarized in tables S4, S5, S6, S7, S8, S9.

### Results for a developed country (DC)

The baseline epidemic curves for both a DC and a LDC for 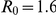 are plotted in red in figure 1. Both countries have similar epidemic curves in that there is no substantial spread before day 60, and the exponential phase of the epidemic starts around day 45. The peak for the LDC occurs slightly earlier than for the DC.

**Figure 2:**
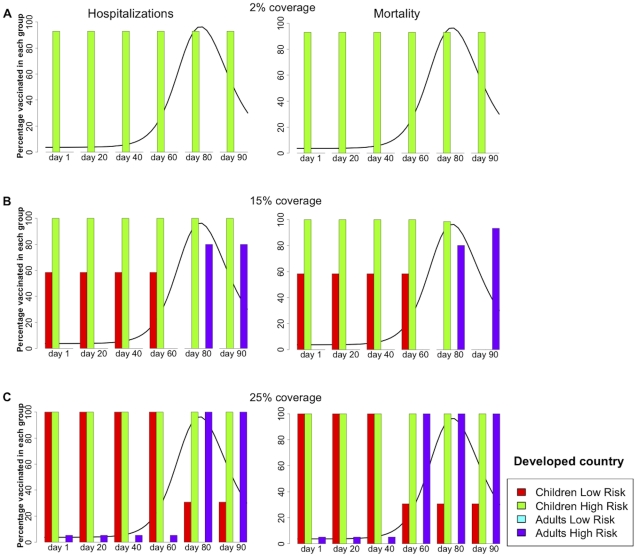
Optimal vaccination policy for a developed country. **Caption:** Optimal vaccination policy when there is enough vaccine to cover (A) 2%, (B) 15%, or (C) 25% of the population in a developed country starting one, 20, 40, 60, 80, or 90 days after the beginning of transmission. The epidemic curve for the symptomatic infections (without any intervention) is shown in black. The left panel minimizes the number of hospitalizations, while the right panel minimizes the number of deaths.

Figure 2 and table 1 summarize the results for the DC population. For each vaccination coverage (figure 2**A**–2**C**), the figure shows the optimal vaccine allocation if vaccination were to start one, 20, 40, 60, 80, or 90 days after the beginning of transmission, both for minimizing hospitalizations (left panel) and mortality (right panel). When there is enough vaccine to cover only two percent of the DC population, the best strategy in both cases is to allocate all the vaccine to the high-risk children (93% coverage) regardless of when vaccination begins (see figure 2).

**Table 1:** Results for a Developed Country (DC). Here, 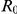 = 1.6.

DC		Day 1	Day 20	Day 40	Day 60	Day 80	Day 90
2% coverage	Optimal strategy (hospitalizations)	[0 93 0 0]^a^	[0 93 0 0]	[0 93 0 0]	[0 93 0 0]	[0 93 0 0]	[0 93 0 0]
	Illness Attack Rate (%)	24.6	24.6	24.7	25.8	27.2	27.5
	Hospitalizations (per 100 cases)	0.3896	0.3898	0.3929	0.4203	0.4485	0.4523
	Optimal strategy (deaths)	[0 93 0 0]	[0 93 0 0]	[0 93 0 0]	[0 93 0 0]	[0 93 0 0]	[0 93 0 0]
	Illness Attack Rate (%)	24.6	24.6	24.7	25.8	27.2	27.5
	Deaths (per 1000 cases)	0.1723	0.1723	0.1726	0.1757	0.1790	0.1795
15% coverage	Optimal strategy (hospitalizations)	[58 100 0 0]	[58 100 0 0]	[58 100 0 0]	[58 100 0 0]	[0 100 0 80]	[0 100 0 80]
	Illness Attack Rate (%)	0.019	0.3	3.8	16.5	26.5	27.2
	Hospitalizations (per 100 cases)	0.4234	0.4237	0.4277	0.4433	0.4370	0.4478
	Optimal strategy (deaths)	[58 100 0 0]	[58 100 0 0]	[58 100 0 0]	[58 100 0 0]	[0 98 0 80]	[0 0 0 93]
	Illness Attack Rate (%)	0.019	0.3	3.8	16.5	26.5	27.2
	Deaths (per 1000 cases)	0.1968	0.1948	0.1924	0.1843	0.1698	0.1753
25% coverage	Optimal strategy (hospitalizations)	[100 100 0 5]	[100 100 0 5]	[100 100 0 5]	[100 100 0 5]	[30 100 0 100]	[30 100 0 100]
	Illness Attack Rate (%)	0.005	0.08	1.5	13.3	25.6	26.8
	Hospitalizations (per 100 cases)	0.4534	0.4528	0.4528	0.4532	0.4395	0.4487
	Optimal strategy (deaths)	[100 100 0 5]	[100 100 0 5]	[100 100 0 5]	[30 100 0 100]	[30 100 0 100]	[30 100 0 100]
	Illness Attack Rate (%)	0.005	0.08	1.5	17.7	25.6	26.8
	Deaths (per 1000 cases)	0.2006	0.1934	0.1920	0.1366	0.1702	0.1759

a [0 93 0 0] denotes the percentages of people vaccinated in each class, where the first entry corresponds to children low-risk, the second one to children high-risk, the third one to adults low-risk and finally adults high-risk.

**Figure 3:**
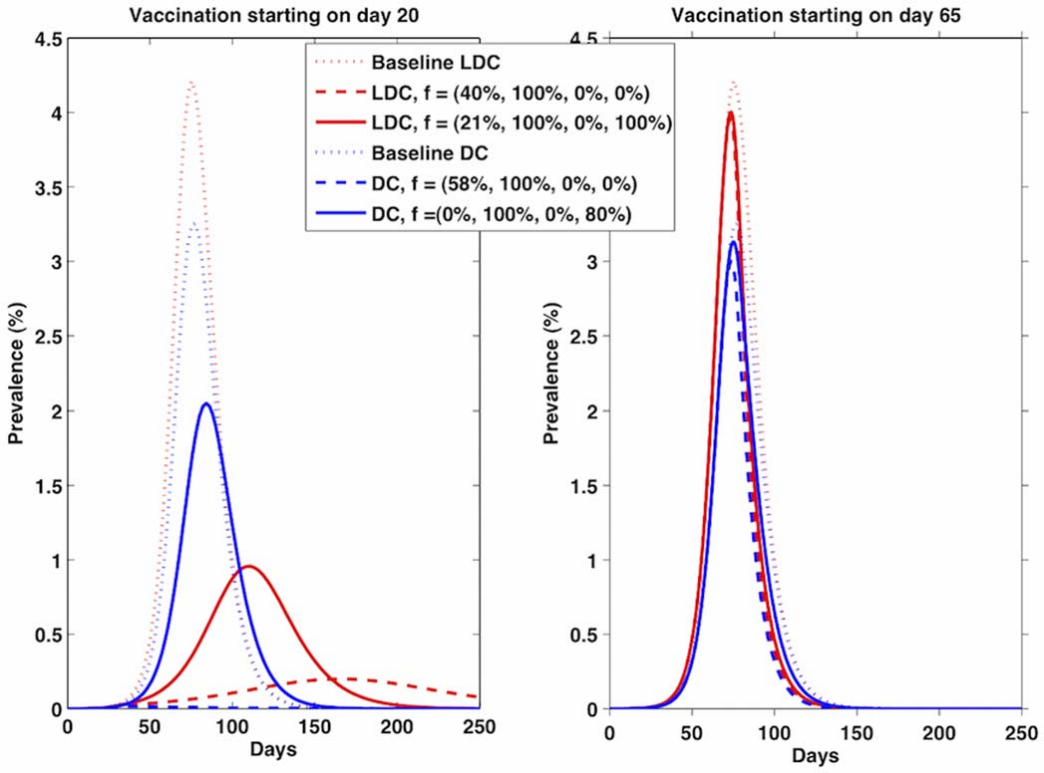
Epidemic curves for a developed country and for a less developed country at two different times. **Caption:** Epidemic curves for a developed country (DC) and for a less developed country (LDC) with vaccination starting at two different times: on day 20 (left panel) and on day 65 (right panel) if we had enough vaccine to cover 15% of the population. The dotted lines represent the baseline epidemic curves for a DC and LDC with no intervention. The dashed lines represent the epidemic curve where we vaccinated all the high-risk children (100%) and used the remainder vaccine in low-risk children (40% in LDC and 58% in DC), while the solid lines represent the epidemic curve corresponding to vaccinate all high-risk children (100%) and high-risk adults (100% in LDC and 80% in DC). For day 20, the optimal vector 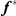, favoring children, (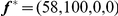 for a DC and 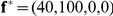 for a LDC) mitigates the epidemic while the other strategy only reduces it. By day 65, both strategies perform poorly.

When supplies are large enough to vaccinate 15% of the population (figure 2**B**), the optimal strategy to reduce both hospitalizations and mortality is to vaccinate all of the high-risk children and then to concentrate the remainder of the vaccine in low-risk children, provided that vaccination occurs before the peak. However, after the peak, it is optimal to cover all high-risk children and to give the remaining vaccine to high-risk adults (this accounts for 80% coverage in this group). For instance, if vaccination were to start 20 days after the beginning of transmission, it would be optimal to give vaccine to all the children at high risk (100% coverage in this group) and to allocate the rest to the children at low risk (58% coverage in this group), but if vaccination were to start 80 days after the beginning of transmission, then it would be optimal to still vaccinate all the high-risk children but to vaccinate a fraction of the high-risk adults (80% coverage in this group). By day 90, it is better to allocate all resources to high-risk adults if minimizing mortality. However, it is important to note that once that the peak of the epidemic has occurred, vaccination has a minimal effect and both strategies mentioned above perform equally poorly (figure 3).

Figure 2**C** presents the results for the DC when enough vaccine is available to cover 25% of the population. Assuming that vaccination occurred before the exponential phase of the epidemic, vaccinating all of the high-risk children and then concentrating the remainder of the vaccine in low-risk children (90% coverage in this group) is the optimal solution, but this time a small amount of vaccine can be given to high-risk adults (19% coverage in high risk adults). In contrast, if vaccination takes place during or after the exponential phase, then it is optimal to favor first all of the high-risk children and all high-risk adults, and then to concentrate the remainder of supplies in low-risk children (30% coverage in low-risk children). For this coverage, the switch in the optimal allocation occurs earlier on if minimizing mortality: By day 80, is better to vaccinate all high-risk people and use the remainder in low-risk children (30% coverage of this group) rather than completely protecting all children and allocate the remainder for high-risk adults.

### Results for a less developed country (LDC)

#### Mortality and hospitalizations unadjusted

**Figure 4:**
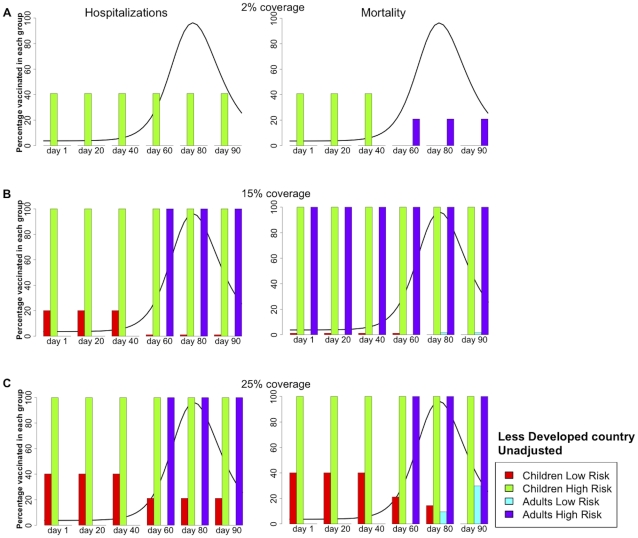
Optimal vaccination policy for a less developed country, unadjusted. **Caption:** Optimal vaccination policy when there is enough vaccine to cover (A) 2%, (B) 15%, or (C) 25% of the population in a less developed country starting one, 20, 40, 60, 80, or 90 days after the beginning of transmission. The epidemic curve for the symptomatic infections (without any intervention) is shown in black. Here, the rates for influenza-related mortality and hospitalizations in a less developed country are exactly the same as those in a developed country (see text S1). The left panel minimizes the number of hospitalizations, while the right panel minimizes the number of deaths.

Figure 4 and table 2 show the analogous results for a LDC, where the hospitalizations and mortality rates were considered to be equal to those in a DC. In this scenario, children make up a much larger proportion of the population (55%) than they do in the DC (24%). When minimizing hospitalizations for very low coverage (2% of the population), it is always optimal to allocate all of the vaccine to high-risk children. However, when minimizing mortality and when vaccination were to occur before the exponential phase, it is optimal to concentrate all of the available vaccine in high-risk children (41% coverage); whereas if vaccination were to occur during or after the exponential phase, it is optimal to shift vaccine coverage to high-risk adults (21% coverage), see figure 4**A**.

**Table 2:** Results for a Less Developed Country (LDC), influenza-related mortality and hospitalizations unadjusted, 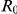 = 1.6.

LDC (unadjusted)		Day 1	Day 20	Day 40	Day 60	Day 80	Day 90
2% coverage	Optimal strategy (hospitalizations)	[0 41 0 0]	[0 41 0 0]	[0 41 0 0]	[0 41 0 0]	[0 41 0 0]	[0 41 0 0]
	Illness Attack Rate (%)	32.7	32.7	32.8	34	34.4	34.5
	Hospitalizations (per 100 cases)	0.3979	0.3981	0.4004	0.4213	0.4438	0.4470
	Optimal strategy (deaths)	[0 41 0 0]	[0 41 0 0]	[0 41 0 0]	[0 0 0 21]	[0 0 0 21]	[0 0 0 21]
	Illness Attack Rate (%)	32.7	32.7	32.8	34	34.4	34.5
	Deaths (per 1000 cases)	0.1127	0.1127	0.1130	0.1139	0.1180	0.1189
15% coverage	Optimal strategy (hospitalizations)	[20 100 0 0]	[20 100 0 0]	[20 100 0 0]	[1 100 0 100]	[1 100 0 100]	[1 100 0 100]
	Illness Attack Rate (%)	19.3	19.5	20.4	29.4	33.4	34.2
	Hospitalizations (per 100 cases)	0.3290	0.3296	0.3380	0.3529	0.4313	0.4460
	Optimal strategy (deaths)	[1 100 0 100]	[1 100 0 100]	[1 100 0 100]	[1 100 0 100]	[0 100 2 100]	[0 100 2 100]
	Illness Attack Rate (%)	26.1	26.1	26.4	29.4	33.4	34.2
	Deaths (per 1000 cases)	0.0599	0.0600	0.0625	0.0851	0.1119	0.1165
25% coverage	Optimal strategy (hospitalizations)	[40 100 0 0]	[40 100 0 0]	[40 100 0 0]	[21 100 0 100]	[21 100 0 100]	[21 100 0 100]
	Illness Attack Rate (%)	1.25	5.3	10.2	24.7	32.6	33.8
	Hospitalizations (per 100 cases)	0.3378	0.3422	0.3581	0.3706	0.4354	0.4438
	Optimal strategy (deaths)	[40 100 0 0]	[40 100 0 0]	[40 100 0 0]	[21 100 0 100]	[14 100 10 100]	[0 100 30 100]
	Illness Attack Rate (%)	1.25	5.3	10.21	24.72	32.7	33.9
	Deaths (per 1000 cases)	0.1141	0.1169	0.1187	0.0905	0.1129	0.1166

When there is enough vaccine for 15% of the population, the results are quite different for minimizing mortality or hospitalizations. For the former, regardless of the phase of the epidemic, it is optimal to protect both the high-risk groups, children and adults, and to allocate the remainder of supplies in low-risk children (1% coverage in this group) before the peak, or low risk adults after the peak, see figure 4**B**. This is because, with this coverage, we would not be able to block transmission by protecting the high-transmission groups. So it is better to directly protect all the members of the most vulnerable groups, and by doing so the number of deaths are greatly diminish. For minimizing hospitalizations, vaccinating low-risk children earlier in the epidemic instead of high-risk adults is better, but later on protecting the high-risk groups is optimal (figure 4**B**).

With enough vaccine to protect 25% of the population, the optimal solutions for minimizing mortality and hospitalizations are identical if vaccination were to occur before the peak of the epidemic. In this case, it is optimal to concentrate vaccine in children (100% of the high-risk children and 40% of the low-risk), see figure 4**C**. But when vaccination occurs later on, the optimal strategy shifts to the high-risk groups, (100% coverage of both children and adults at high-risk) with allocation of the remainder of the vaccine to low-risk children (21% coverage in this group) to minimize hospitalizations, or to low-risk adults when minimizing mortality.

#### Adjusted mortality and hospitalizations

**Figure 5:**
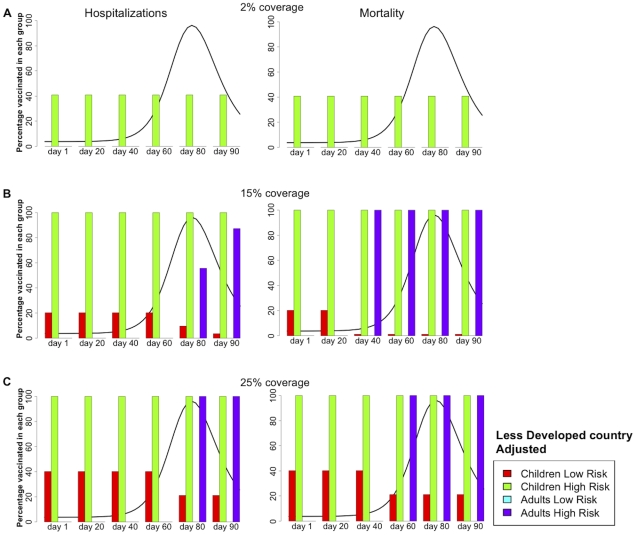
Optimal vaccination policy for a less developed country, adjusted. **Caption:** Optimal vaccination policy when there is enough vaccine to cover (A) 2%, (B) 15%, or (C) 25% of the population in a less developed country starting one, 20, 40, 60, 80, or 90 days after the beginning of transmission. The epidemic curve for the symptomatic infections (without any intervention) is shown in black. Here, the model was adjusted to account for the excess in mortality and hospitalizations in a less developed country compared with a developed country (see text S1). The left panel minimizes the number of hospitalizations, while the right panel minimizes the number of deaths.

Figure 5 and table 3 summarize the results for a LDC where we adjusted for the excess in mortality and hospitalizations. This leads to more uniform policies in which children tend to get vaccinated. For low coverage, the optimal policy is always, for all times considered, to cover the high-risk children (see figure 5**A**). As coverage increases, it is optimal to first protect high-risk children, secondly, to allocate the remainder of the resources either to low-risk children or to high-risk adults and then to allocate any extra vaccine to low-risk children. The former is optimal before the exponential phase of the epidemic, while the latter is better if vaccination starts later on (see figure 5**B**). Minimizing the number of deaths tends to move the threshold for protecting high-risk adults over low-risk children to the left. For example, when there is enough vaccine to cover 25% of the population, it is optimal to cover high-risk adults over low-risk children if vaccination were to start at day 60 and we wanted to avert deaths. However the opposite would hold if we wanted to avert hospitalizations (figure 5**C**).

**Table 3:** Results for a Less Developed Country (LDC), influenza-related mortality and hospitalizations adjusted, 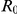 = 1.6.

LDC (adjusted)		Day 1	Day 20	Day 40	Day 60	Day 80	Day 90
2% coverage	Optimal strategy (hospitalizations)	[0 41 0 0]	[0 41 0 0]	[0 41 0 0]	[0 41 0 0]	[0 41 0 0]	[0 41 0 0]
	Illness Attack Rate (%)	32.7	32.8	32.8	33.5	34.4	34.5
	Hospitalizations (per 100 cases)	2.5093	2.5103	2.5293	2.7016	2.8858	2.9119
	Optimal strategy (deaths)	[0 41 0 0]	[0 41 0 0]	[0 41 0 0]	[0 41 0 0]	[0 41 0 0]	[0 41 0 0]
	Illness Attack Rate (%)	32.7	32.8	32.8	33.5	34.4	34.5
	Deaths (per 1000 cases)	0.5308	0.5310	0.5337	0.5584	0.5849	0.5886
15% coverage	Optimal strategy (hospitalizations)	[20 100 0 0]	[20 100 0 0]	[20 100 0 0]	[20 100 0 0]	[10 100 0 55]	[10 100 0 55]
	Illness Attack Rate (%)	19.3	19.5	20.4	26.8	33.3	34.1
	Hospitalizations (per 100 cases)	1.8765	1.8804	1.9545	2.4732	2.8463	2.8956
	Optimal strategy (deaths)	[20 100 0 0]	[20 100 0 0]	[1 100 0 100]	[1 100 0 100]	[1 100 0 100]	[1 100 0 100]
	Illness Attack Rate (%)	19.3	19.5	26.4	29.4	33.4	34.4
	Deaths (per 1000 cases)	0.4568	0.4568	0.3356	0.4463	0.5627	0.5801
25% coverage	Optimal strategy (hospitalizations)	[40 100 0 0]	[40 100 0 0]	[40 100 0 0]	[40 100 0 0]	[21 100 0 100]	[21 100 0 100]
	Illness Attack Rate (%)	1.25	5.3	10.2	22.6	32.6	33.8
	Hospitalizations (per 100 cases)	1.8984	1.9066	2.0416	2.5836	2.8620	2.9040
	Optimal strategy (deaths)	[40 100 0 0]	[40 100 0 0]	[40 100 0 0]	[21 100 0 100]	[21 100 0 100]	[21 100 0 100]
	Illness Attack Rate (%)	1.25	5.3	10.2	24.7	32.6	33.8
	Deaths (per 1000 cases)	0.4711	0.4789	0.4972	0.4711	0.5686	0.5823

### Sensitivity analysis

Choosing the optimal strategy is extremely important before the peak of the epidemic, but once we reach it, vaccination has little effect and all strategies perform poorly. Figure 3 shows the epidemic curves for a DC and for a LDC with vaccination starting at two different times: On day 20 (left panel) and on day 65 (right panel) if we had enough vaccine to cover 15% of the population in each country. In each panel, we plotted two strategies: the optimal strategy for day 20 (this is, vaccinating 40% of the low-risk children and 100% of the high-risk children) and the optimal strategy for day 65 (this corresponds to vaccinating 21% of the low-risk children, and fully protect the high-risk groups). Choosing the optimal strategy if vaccination starts on day 20 mitigates the epidemic for the DC, and gives a very mild epidemic in the LDC, while vaccinating the high-risk people only reduces the size of the epidemic in both countries. However, by day 65, both policies give similar epidemic curves that almost overlay with the baseline curve.

If vaccination occurs early in the epidemic or the coverage is very low (2%), then the optimal vaccine allocation is not sensitive to changes in the basic reproduction number 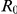. However, when vaccine coverage increases, then the optimal strategy to minimize deaths, and to a lesser extent, to minimize hospitalizations, shifts from low-risk children to high-risk adults faster as 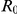 increases (see figure S2).

**Figure 6:**
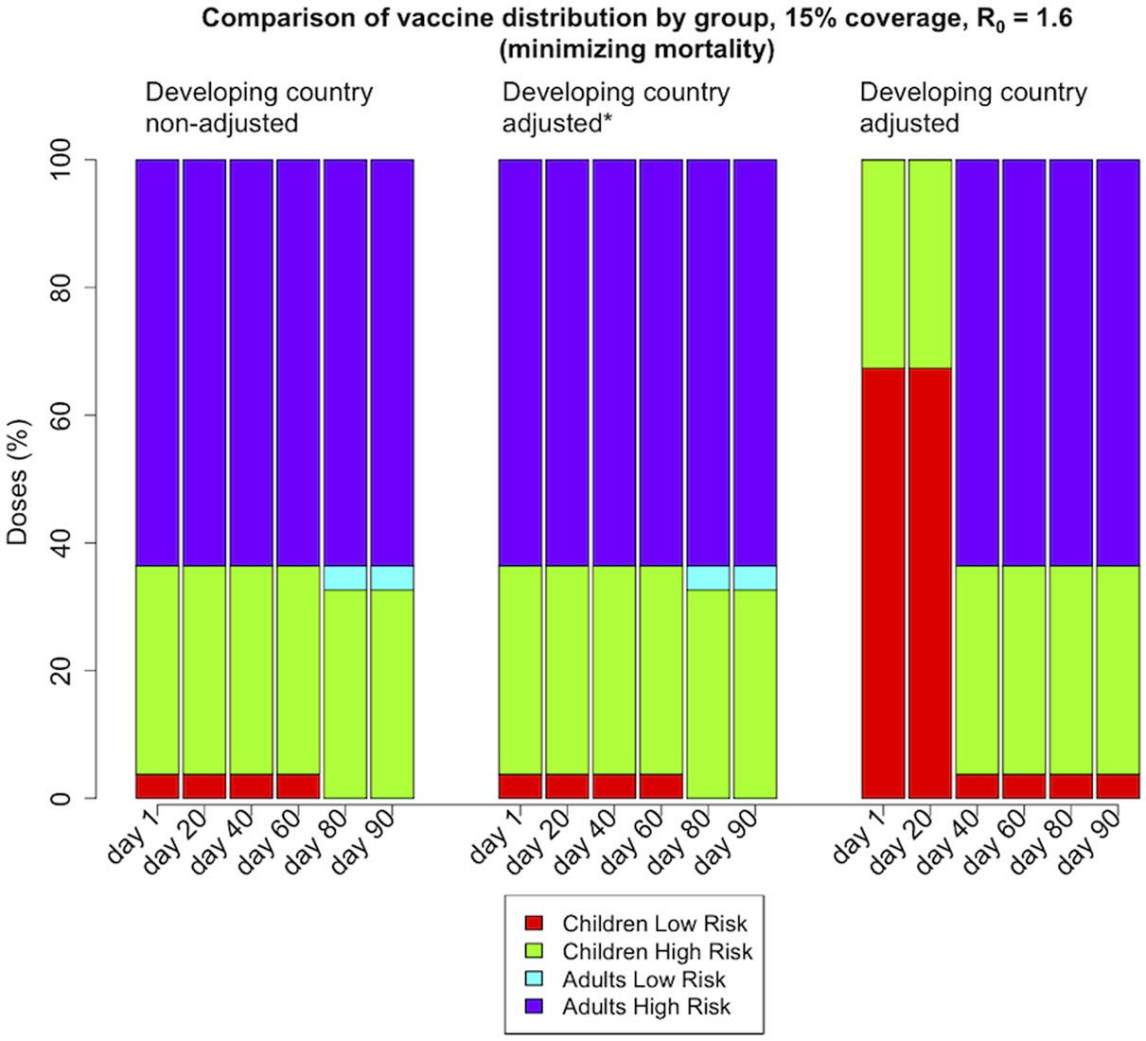
Sensitivity analysis for adjusting influenza-related mortality and hospitalizations in a developing country setting. **Caption:** Percentage of the total number of doses used in each sub-group in a less developed country when there is enough vaccine to protect 15% of the population and the objective function was set to minimize mortality. The left panel shows the optimal values without adjusting for the excess of deaths and hospitalizations, while in the right panel we adjusted these parameters by multiplying them by an adjusting factor (see text S1 and table S2). The middle panel shows an intermediate adjustment: Here the adjusting factors were taken as half of the ones given in table S2 (increase in the influenza-related mortality by a factor of 4 instead of 8 in children and 1.5 instead of 3 in adults). Adjusting these numbers tends to favor the protection of low-risk children early in the epidemic, as opposed to protect high-risk adults.

The model is very sensitive to the parameters for excess of deaths and hospitalizations in less developed countries. In order to investigate this, we repeated the analysis and halved the values of each of these parameters i.e., the influenza-related mortality and hospitalizations were increased by a factor of four instead of a factor of eight in children and by 1.5 instead of three in adults. Figure 6 shows the percentage of the total number of doses used in each group when there is enough vaccine to protect 15% of the population and the optimization was set to minimize mortality. When mortality and hospitalizations are not adjusted, the model favors the high-risk groups, especially early on in the epidemic. As we increase the multipliers adjusting for these parameters, the optimal solution shifts to favor the low-risk children instead. Varying the proportions of high-risk people, the mortality and the hospitalization rates in each group greatly changes the optimization policies. This is expected as these numbers determine the outcome of the optimization directly. Augmenting these parameters in a given group will result in an optimal strategy where that group tends to be favored (see Text S1 and figures S3 and S4).

## Discussion

We use a mathematical model to find the optimal vaccine allocation at different time points of an epidemic. For both developed and less developed countries, when faced with low supplies of vaccines, it is always optimal to concentrate vaccine in high-risk children to provide them with direct protection, as they are part of the high-transmission chain and they are among the most vulnerable.

For a developed country, as vaccine supplies increases, it becomes optimal to allocate the resources in the high-transmission group, i.e., children at low-risk. This makes sense, since by protecting the high-transmission group, we stop the chain of transmission and indirectly protect the high risk groups. However, this policy is optimal only up to a certain time during or after the exponential rise phase of the epidemic, when too many high-transmission people have already been infected and have acquired natural immunity. After this point in time, it is optimal to concentrate vaccine in high-risk groups protecting them directly. Minimizing mortality, as opposed to hospitalizations, tends to push this threshold to the left in time so that the protection of high-risk adults starts earlier.

This is also true for less developed countries when minimizing hospitalizations. In contrast, when averting deaths, it is better to allocate vaccine in the high-risk groups first and then cover high-transmission groups. Once vaccine supplies reach a certain coverage level, then it becomes important to vaccinate the high-transmission groups in the earlier stages of the epidemic, but this policy becomes suboptimal once the peak of the epidemic has passed. This is because by allocating this much vaccine in children earlier on in the epidemic, we would be able to block transmission and mitigate the disease, but if vaccination took place later on in the epidemic, there are too many people already infected and this strategy is no longer optimal (see figure 3).

These results highlight several important components of influenza epidemic control with vaccines. First, the proportion of population that is children is extremely important. For a less developed country, where the high-transmission group accounts for the majority of the population, one needs large amounts of vaccine to indirectly protect the high-risk groups by vaccinating the high-transmission ones. However, in a developed country, where high-risk groups represent a smaller fraction of the population, it is possible to reduce and even mitigate transmission by vaccinating the high-transmission groups, if this is done early in the epidemic. The second important point is that timing of the vaccination is extremely important and greatly determines where the efforts should be concentrated. Finally, while using the optimal policy greatly reduces the size of the epidemic if done early on, all vaccination policies perform poorly after the peak of the epidemic. It is very difficult to identify in real time where one is in an epidemic, but it would be even more difficult to switch the vaccination target groups during an epidemic, both politically and logistically. Given that timing is crucial, this suggests the necessity of better surveillance and preparedness. In this context, our results could be used to set general a priori guidelines for vaccine distribution on a given population. For example, pandemic H1N1 peaked in the US in early October 2009, and during the same period vaccination with limited supplies of vaccine began. As a result, most vaccine was delivered and administered well after the peak. The mass vaccination of children that occurred had a minimal effect on protecting others and reducing general morbidity in the population. Despite the early accurate prediction of when the epidemic would peak in the US [22], the vaccine arrived during the peak, for logistical reasons, and had a limited effect.

As a first approach, we used excess in infant mortality and excess in female adult mortality as proxies for excess in influenza-related mortality and hospitalizations in a LDC compared with a DC. The results were very sensitive to these parameters. This suggests that we need studies to more accurately determine these numbers. Hospitalizations and mortality rates in a LDC are very different from those in a DC. People tend to not seek medical attention unless strictly necessary due to the lack of health insurance and the economic cost. In a pandemic situation, the health system in a LDC is likely to quickly run out of essential medications, to lack essential health personnel or to reach full capacity. This will in turn increase mortality. None of these factors were considered in our model. Furthermore, our results are extremely sensitive to the population structure, both in the percentages of people at high risk and in the contact pattern among them. Here, we assumed that the contact patterns were identical in a DC and a LDC. This is an important limitation since the model depends strongly in this assumption. Given the uncertainty for the parameters for pandemic H1N1 2009, we agree with Dushoff et al. [29] that one should be cautious in interpreting the results offered by simple models.

The model presented here is extremely simple. While we are able to draw general conclusions, our results may not be appropriate for specific countries. Adding more structure to the model (for example adding more age groups, changing the probabilities of transmission in each group, and adding other details) will make a more realistic model for a specific situation, and hence more realistic predictions. We assumed that the vaccine efficacy was the same in all groups. This is a limitation since we know that the efficacy is reduced in the elderly and takes more time to develop in children. Finally one could expect somewhat different results if the objective function were replaced by other functions, such as final illness attack rates, remaining years of life lost, economic burden or a combination of these.

Previous work [30], [31], [32] has suggested that in presence of low vaccine supplies, high-risk groups should be prioritized but high-transmission groups should be vaccinated with larger quantities of vaccine. Our results agree with this strategy for a population with a structure similar to the one in the US as long as vaccination starts before the peak of the epidemic. However, we suggest that there is a threshold in the time when a switch in the optimal strategy occurs, after which, vaccine would be more effective if allocated directly to the high-risk groups. This is in agreement with the results found by others [9], [33], [34]. The particular time for this threshold is strongly dependent on the values of the model parameters, in particular on the vaccination coverage and population structure, but in general, occurs some time during the exponential phase of the epidemic or right at the peak. Our results suggest that if vaccination occurs too close to the peak of the epidemic, then all the strategies considered performed poorly, in agreement with recent work [11], [12]. Our results are novel in that we compared optimal strategies for both a developed country and a developing country, taking into account differences in the population structure and excess in influenza-related mortality and hospitalizations.

## Supporting Information

Figure S1 **Vaccine efficacy as a function of time.** Plot of the vaccine efficacies modeled as functions of time. Once vaccine is administered, the vaccine efficacies build up in time in an exponentially-like fashion during the first 15 days and remain constant afterward. The exact formula is given in Text S1.
(0.02 MB PDF)

Figure S2 **Sensitivity analysis for the basic reproduction number R_0_ for a developed country.** Vaccine distribution by group, for a developed country, with vaccine enough to cover 15% of the population, minimizing deaths for R_0_ = 1.4, R_0_ = 1.6 and R_0_ = 1.8 and set of respective dates considered. As R_0_ increases, the optimal solution shifts the tiping point where there is a switch from protecting low-risk children to high-risk adults.
(0.05 MB PDF)

Figure S3 **Sensitivity analysis for adjusting influenza-related mortality and hospitalizations in a LDC setting.** Percentage of the total number of doses used in each sub-group in a less developed country when there is enough vaccine to protect 15% of the population and the objective function was set to minimize hospitalizations. The left panel shows the optimal values without adjusting for excess of deaths and hospitalizations, while in the right panel we adjusted these parameters by multiplying them by an adjusting factor (see text and table S2). The middle panel illustrates a middle-ground adjustment: The multipliers given in table S2 were halved. (increase in the influenza-related mortality by a factor of 4 instead of 8 in children and 1.5 instead of 3 in adults).
(0.06 MB PDF)

Figure S4 **Sensitivity analysis for adjusting influenza-related mortality and hospitalizations in a LDC setting.** Percentage of the total number of doses used in each sub-group in a less developed country when there is enough vaccine to protect 25% of the population and the objective function was set to minimize mortality. The left panel shows the optimal values without adjusting for excess of deaths and hospitalizations, while in the right panel we adjusted these parameters by multiplying them by an adjusting factor (see text and table S2). The middle panel illustrates a middle-ground adjustment: the multipliers given in table S2 were halved (increase in the influenza-related mortality by a factor of 4 instead of 8 in children and 1.5 instead of 3 in adults).
(0.06 MB PDF)

Table S1 **Times considered for starting vaccination for each R_0_.**
(0.03 MB PDF)

Table S2 **Parameter values.**
(0.06 MB PDF)

Table S3 **Final illness attack rates for the developed country setting for the range of basic reproduction numbers considered.**
(0.03 MB PDF)

Table S4 **Results for a Developed Country with R_0_ = 1.4.**
(0.09 MB PDF)

Table S5 **Results for a Developed Country with R_0_ = 1.8.**
(0.08 MB PDF)

Table S6 **Results for a Less Developed Country, influenza-related mortality and hospitalizations unadjusted, R_0_ = 1.4.**
(0.07 MB PDF)

Table S7 **Results for a Less Developed Country, influenza-related mortality and hospitalizations adjusted, R_0_ = 1.4.**
(0.07 MB PDF)

Table S8 **Results for a Less Developed Country, influenza-related mortality and hospitalizations unadjusted, R_0_ = 1.8.**
(0.07 MB PDF)

Table S9 **Results for a Less Developed Country, influenza-related mortality and hospitalizations adjusted, R_0_ = 1.8.**
(0.07 MB PDF)

Text S1 **Supplemental material for ‘Optimizing vaccine allocation at different points in time during an epidemic’.**
(0.11 MB PDF)
