# Supplementary figures and images for: Optimizing Vaccine Allocation at Different Points in Time during an Epidemic
Source: PLoS One. 2010 Nov 11;5(11):e13767. doi: 10.1371/journal.pone.0013767 (PMC2978681; doi:10.1371/journal.pone.0013767)

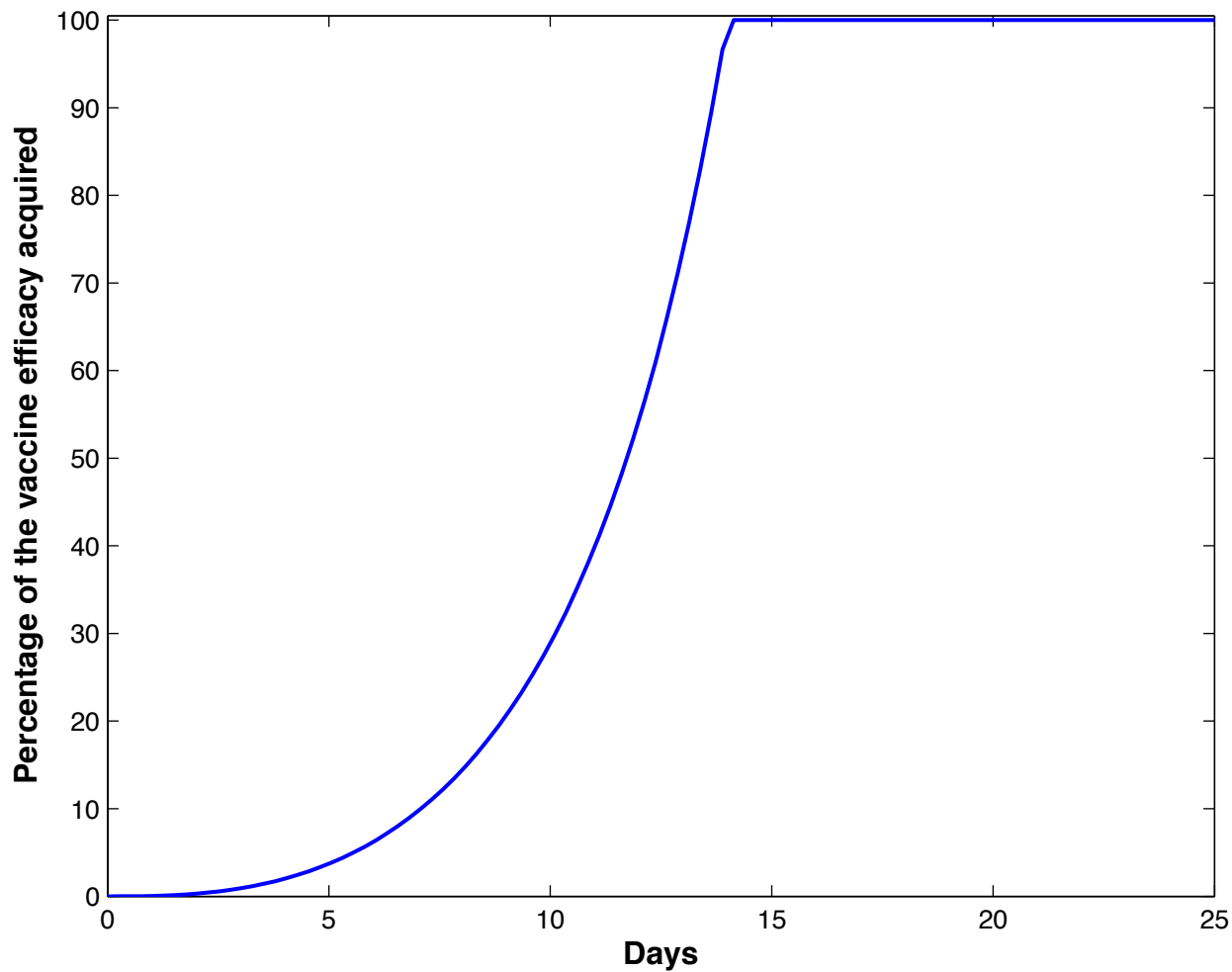

Supplement: Figure S1 — Vaccine efficacy as a function of time. Plot of the vaccine efficacies modeled as functions of time. Once vaccine is administered, the vaccine efficacies build up in time in an exponentially-like fashion during the first 15 days and remain constant afterward. The exact formula is given in Text S1. (0.02 MB PDF) [file pone.0013767.s001.pdf]

**Vaccine distribution by group, 15% coverage**  
**Developed country, minimizing mortality**

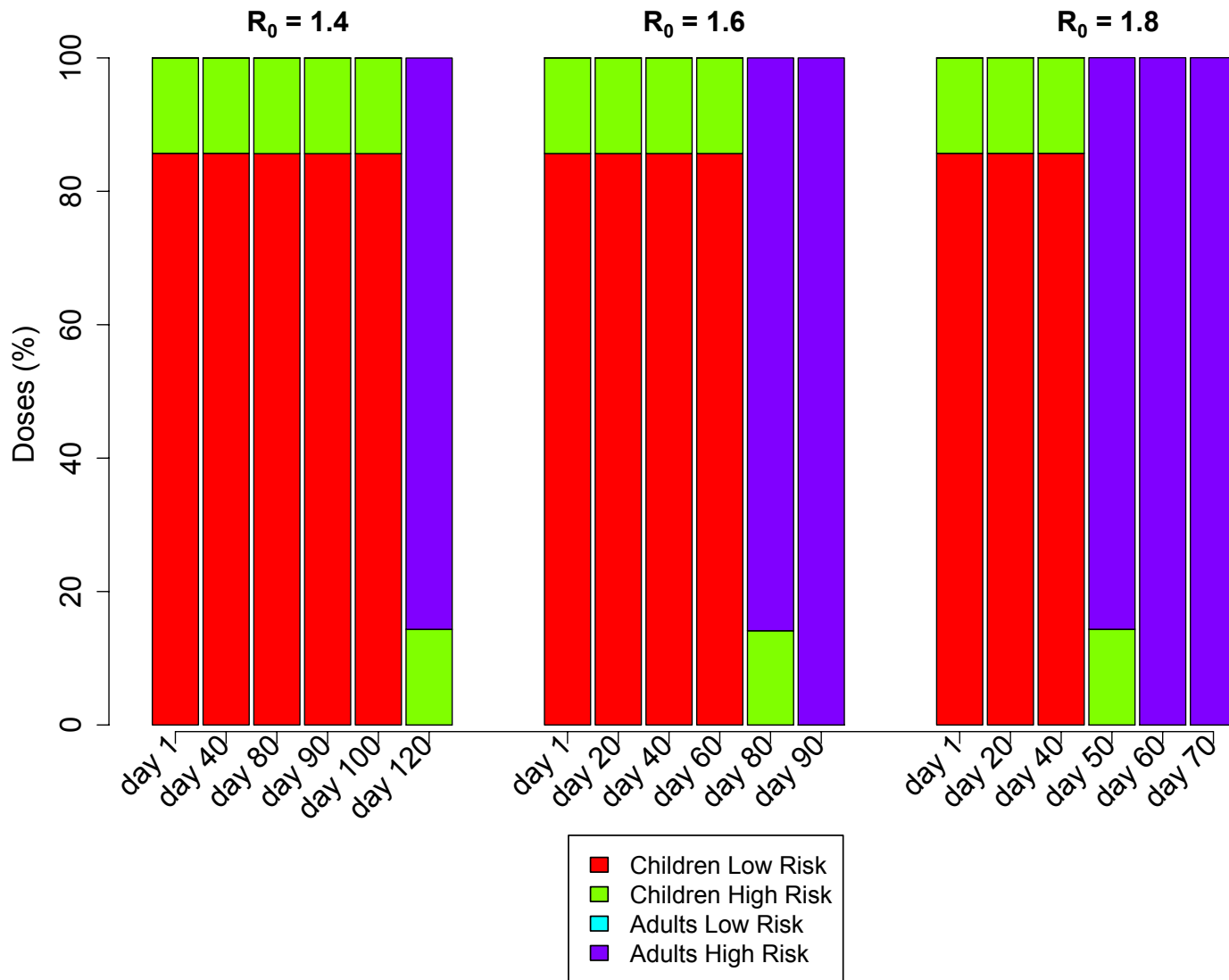

Supplement: Figure S2 — Sensitivity analysis for the basic reproduction number R0 for a developed country. Vaccine distribution by group, for a developed country, with vaccine enough to cover 15% of the population, minimizing deaths for R0 = 1.4, R0 = 1.6 and R0 = 1.8 and set of respective dates considered. As R0 increases, the optimal solution shifts the tiping point where there is a switch from protecting low-risk children to high-risk adults. (0.05 MB PDF) [file pone.0013767.s002.pdf]

**Comparison of vaccine distribution by group, 15% coverage,  $R_0 = 1.6$   
(minimizing hospitalizations)**

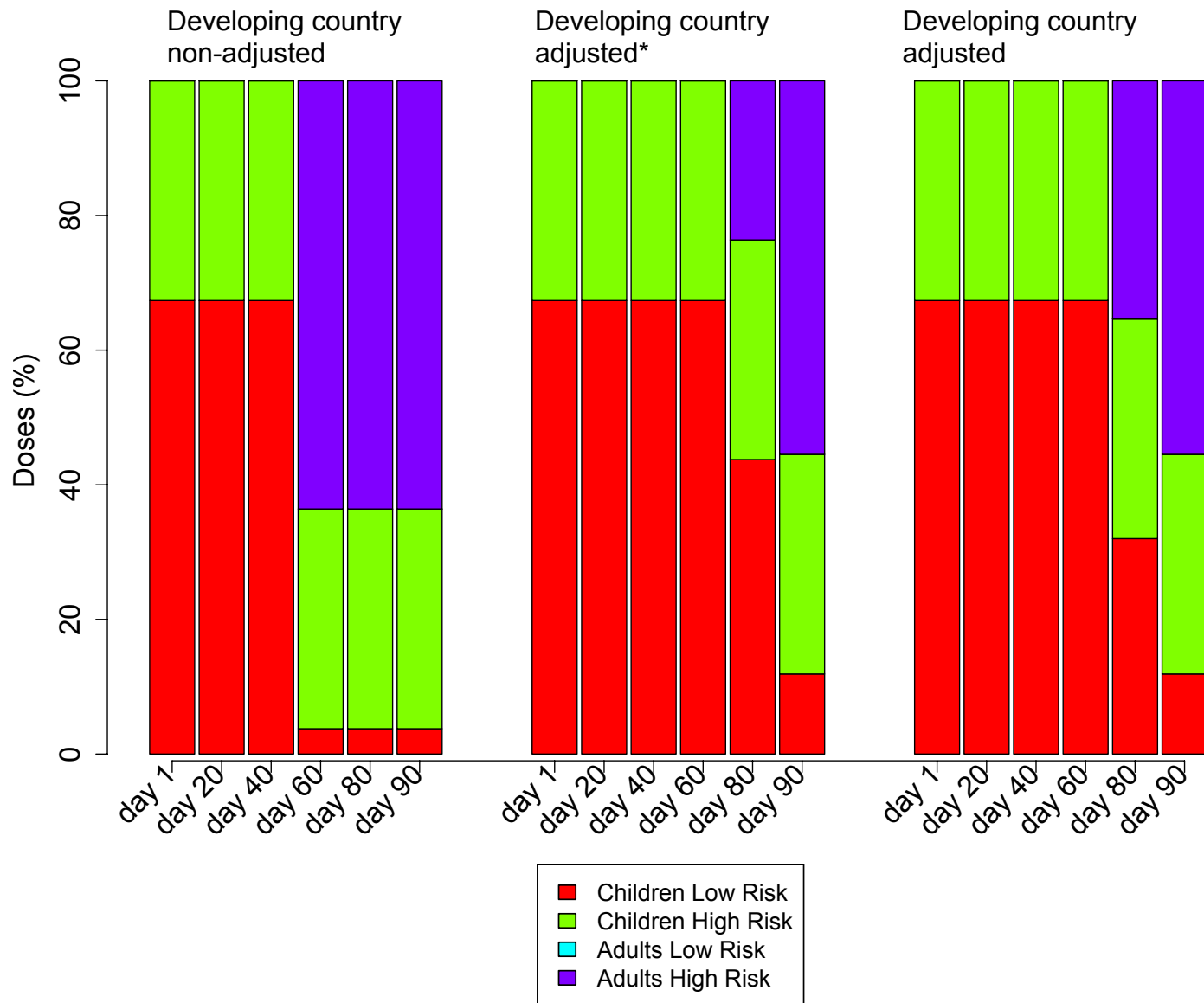

Supplement: Figure S3 — Sensitivity analysis for adjusting influenza-related mortality and hospitalizations in a LDC setting. Percentage of the total number of doses used in each sub-group in a less developed country when there is enough vaccine to protect 15% of the population and the objective function was set to minimize hospitalizations. The left panel shows the optimal values without adjusting for excess of deaths and hospitalizations, while in the right panel we adjusted these parameters by multiplying them by an adjusting factor (see text and table S2). The middle panel illustrates a middle-ground adjustment: The multipliers given in table S2 were halved. (increase in the influenza-related mortality by a factor of 4 instead of 8 in children and 1.5 instead of 3 in adults). (0.06 MB PDF) [file pone.0013767.s003.pdf]

**Comparison of vaccine distribution by group, 25% coverage,  $R_0 = 1.6$   
(minimizing mortality)**

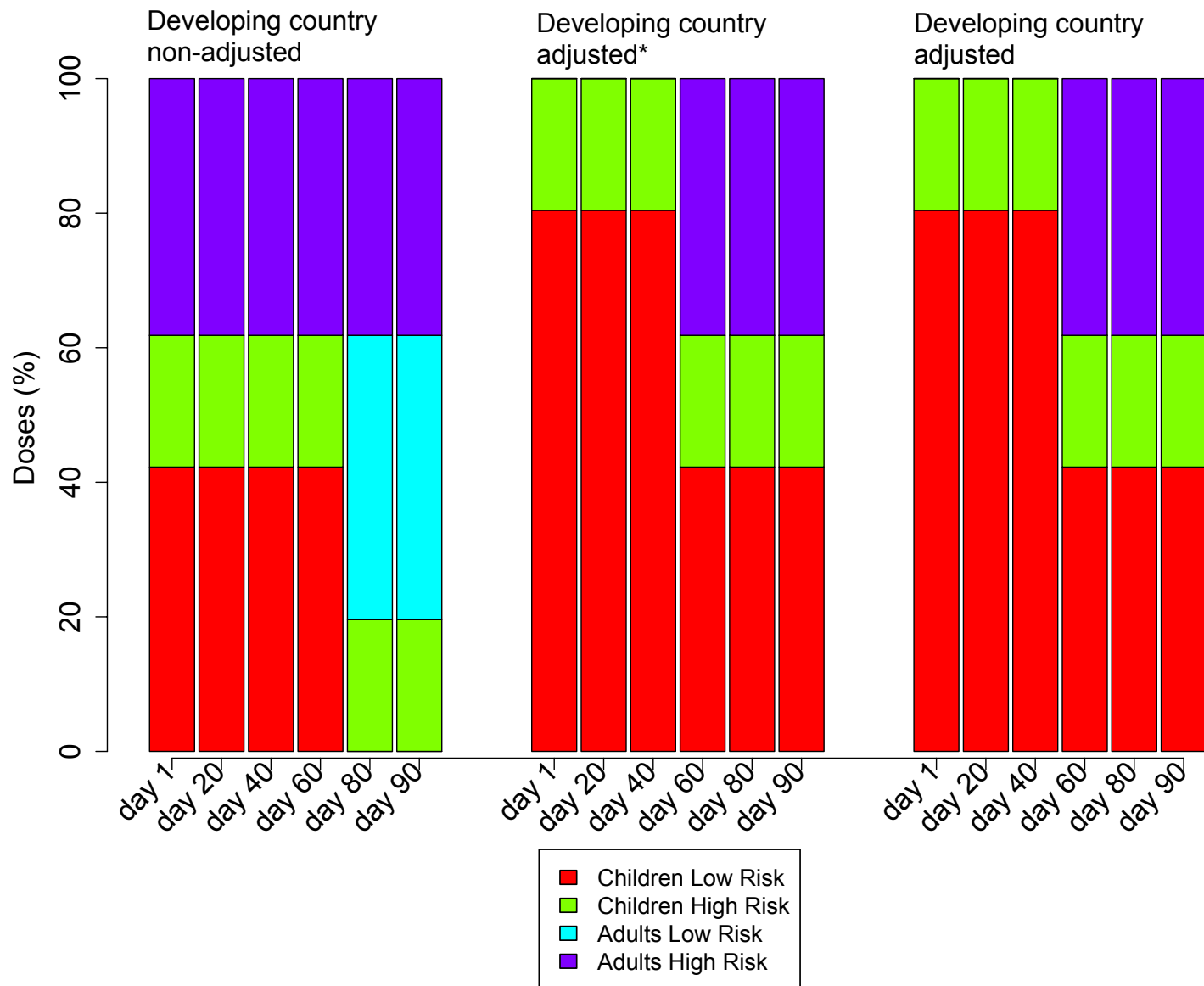

Supplement: Figure S4 — Sensitivity analysis for adjusting influenza-related mortality and hospitalizations in a LDC setting. Percentage of the total number of doses used in each sub-group in a less developed country when there is enough vaccine to protect 25% of the population and the objective function was set to minimize mortality. The left panel shows the optimal values without adjusting for excess of deaths and hospitalizations, while in the right panel we adjusted these parameters by multiplying them by an adjusting factor (see text and table S2). The middle panel illustrates a middle-ground adjustment: the multipliers given in table S2 were halved (increase in the influenza-related mortality by a factor of 4 instead of 8 in children and 1.5 instead of 3 in adults). (0.06 MB PDF) [file pone.0013767.s004.pdf]
