# Supplementary material for: Optimizing Vaccine Allocation at Different Points in Time during an Epidemic
Source: PLoS One. 2010 Nov 11;5(11):e13767. doi: 10.1371/journal.pone.0013767 (PMC2978681; doi:10.1371/journal.pone.0013767)
Supplement: Table S1 — Times considered for starting vaccination for each R0. (0.03 MB PDF) [file pone.0013767.s005.pdf]

Table S1: Times considered for starting vaccination for each R_0.

R_0	Vaccination times					
1.4	1	40	80	90	100	120
1.6	1	20	40	60	80	90
1.8	1	20	40	50	60	70
