# Supplementary material for: Optimizing Vaccine Allocation at Different Points in Time during an Epidemic
Source: PLoS One. 2010 Nov 11;5(11):e13767. doi: 10.1371/journal.pone.0013767 (PMC2978681; doi:10.1371/journal.pone.0013767)
Supplement: Table S2 — Parameter values. (0.06 MB PDF) [file pone.0013767.s006.pdf]

Table S2: Parameter values.

Parameter	Description	Value	Reference
γ	recovery rate	0.25	[14]
ρ	fraction of symptomatic	2/3	[14]
m	reduction of infectiousness for asymptomatics	0.5	[14]
$c_{11}, c_{12}, c_{21}, c_{22}$	contact rates	1,0.2, 0.2, 0.4	calculated[a]
$\mathsf{VE_S}, \mathsf{VE_I}, \mathsf{VE_P}$	vaccine efficacies for susceptibility, infectiousness and pathogenicity	0.4, 0.45, 0.75	[15]
N	total population	200 000	assumption
	initially infected fraction of the population	1	assumption
	percentage of children under 18 (US)	24.16	[16]
	percentage of children under 19 (less developed country)	55	[17]
δ_1	percentage of children at high risk	8.9	[18]
δ_2	percentage of adults at high risk	21.2	[18]
	mortality in low risk children	0.000031	[19][b]
	mortality in high risk children	0.000416	[19]
	mortality in low risk adults	0.000101	[19]
	mortality in high risk adults	0.000821	[19]
	hospitalization rate in low risk children	0.0021	[19][c]
	hospitalization rate in high risk children	0.0279	[19]
	hospitalization rate in low risk adults	0.0024	[19]
	hospitalization rate in high risk adults	0.0129	[19]
	adjusted multiplier for mortality and hospitalizations for children in LDC	8	assumption
	adjusted multiplier for mortality and hospitalizations for adults in LDC	3	assumption

[a] The contact rates were calculated to obtain the final illness attack rates shown in table S3

[b] The mortality rates were computed by weighting the estimates of deaths given in [19] by the percentages of people in each subgroup given in [18].

[c] The hospitalization rates were computed by weighting the estimates of hospitalizations given in [19] by the percentages of people in each subgroup given in [18].
