# Supplementary material for: Optimizing Vaccine Allocation at Different Points in Time during an Epidemic
Source: PLoS One. 2010 Nov 11;5(11):e13767. doi: 10.1371/journal.pone.0013767 (PMC2978681; doi:10.1371/journal.pone.0013767)
Supplement: Table S3 — Final illness attack rates for the developed country setting for the range of basic reproduction numbers considered. (0.03 MB PDF) [file pone.0013767.s007.pdf]

Table S3: Final illness attack rates for the developed country setting for the range of basic reproduction numbers considered.

R_0	Overall illness attack rate	Illness attack rate in children	Illness attack rate in adults
1	0	0	0
1.2	8.1	11.6	3.9
1.3	19.9	27.4	10.6
1.4	26.7	35.9	15.5
1.5	31	40.8	19
1.6	34.6	44.8	22.1
1.7	37.9	48.3	25.3
1.8	40.6	50.9	28
