# Supplementary material for: Optimizing Vaccine Allocation at Different Points in Time during an Epidemic
Source: PLoS One. 2010 Nov 11;5(11):e13767. doi: 10.1371/journal.pone.0013767 (PMC2978681; doi:10.1371/journal.pone.0013767)
Supplement: Table S4 — Results for a Developed Country with R0 = 1.4. (0.09 MB PDF) [file pone.0013767.s008.pdf]

Table S4: Results for a Developed Country with $R_0 = 1.4$.

**Developed Country** $\mathbf{R_0 = 1.4}$		Day 1	Day 40	Day 80	Day 90	Day 100	Day 120
2% coverage	Optimal strategy (hospitalizations)	[0 93 0 0] [a]	[0 93 0 0]	[0 93 0 0]	[0 93 0 0]	[0 93 0 0]	[0 93 0 0]
	Illness Attack Rate (%)	17.1	17.1	17.2	18	19.4	20.3
	Hospitalizations (per 100 cases)	0.3827	0.3828	0.3833	0.4030	0.4333	0.4488
	Optimal strategy (deaths)	[0 93 0 0]	[0 93 0 0]	[0 93 0 0]	[0 93 0 0]	[0 93 0 0]	[0 93 0 0]
	Illness Attack Rate (%)	17.1	17.1	17.2	18	19.4	20.3
	Deaths (per 1000 cases)	0.1652	0.1652	0.1653	0.1677	0.1713	0.1732
15% coverage	Optimal strategy (hospitalizations)	[58 100 0 0]	[58 100 0 0]	[58 100 0 0]	[58 100 0 0]	[58 100 0 0]	[58 100 0 0]
	Illness Attack Rate (%)	0	0.03	0.25	7.2	15.3	19.2
	Hospitalizations (per 100 cases)	0.4309	0.4308	0.4310	0.4398	0.4490	0.4528
	Optimal strategy (deaths)	[58 100 0 0]	[58 100 0 0]	[58 100 0 0]	[58 100 0 0]	[58 100 0 0]	[0 100 0 80]
	Illness Attack Rate (%)	0	0.03	0.25	7.2	15.3	19.2
	Deaths (per 1000 cases)	0.1897	0.1833	0.1822	0.1785	0.1754	0.1672
25% coverage	Optimal strategy (hospitalizations)	[100 100 0 5]	[100 100 0 5]	[100 100 0 5]	[100 100 0 5]	[100 100 0 5]	[38 100 0 90]
	Illness Attack Rate (%)	0.003	0.02	0.14	5.5	14.2	19.2
	Hospitalizations (per 100 cases)						
	Optimal strategy (deaths)	[100 100 0 5]	[100 100 0 5]	[100 100 0 5]	[83 100 0 28]	[30 100 0 100]	[30 100 0 100]
	Illness Attack Rate (%)	0.003 0.02	0.1	5.8	15.7	19.3	
	Deaths (per 1000 cases)	0.1949	0.1831	0.1814	0.1707	0.1536	0.1682

[a] [0 93 0 0] denotes the percentages of people vaccinated in each class, where the first entry corresponds to children low-risk, the second one to children high-risk, the third one to adults low-risk and finally adults high-risk.
