# Supplementary material for: Optimizing Vaccine Allocation at Different Points in Time during an Epidemic
Source: PLoS One. 2010 Nov 11;5(11):e13767. doi: 10.1371/journal.pone.0013767 (PMC2978681; doi:10.1371/journal.pone.0013767)
Supplement: Table S5 — Results for a Developed Country with R0 = 1.8. (0.08 MB PDF) [file pone.0013767.s009.pdf]

Table S5: Results for a Developed Country with $R_0 = 1.8$.

**Developed Country** $\mathbf{R_0 = 1.8}$		Day 1	Day 20	Day 40	Day 50	Day 60	Day 70
2% coverage	Optimal strategy (hospitalizations)	[0 93 0 0]	[0 93 0 0]	[0 93 0 0]	[0 93 0 0]	[0 93 0 0]	[0 93 0 0]
	Illness Attack Rate (%)	30.8	30.9	31.5	33.5	33.5	33.5
	Hospitalizations (per 100 cases)						
	Optimal strategy (deaths)	[0 93 0 0]	[0 93 0 0]	[0 93 0 0]	[0 93 0 0]	[0 93 0 0]	[0 93 0 0]
	Illness Attack Rate (%)	30.8	30.9	31.5	33.5	33.5	33.5
	Deaths (per 1000 cases)	0.1779	0.1780	0.1799	0.1846	0.1846	0.1846
15% coverage	Optimal strategy (hospitalizations)	[58 100 0 0]	[58 100 0 0]	[58 100 0 0]	[0 100 0 80]	[0 100 0 80]	[0 100 0 80]
	Illness Attack Rate (%)	0.4	4.7	17.8	33.4	33.52	33.5
	Hospitalizations (per 100 cases)	0.4205	0.4230	0.4382	0.4532	0.4548	0.4550
	Optimal strategy (deaths)	[58 100 0 0]	[58 100 0 0]	[58 100 0 0]	[58 100 0 0]	[58 100 0 0]	[0 100 0 80]
	Illness Attack Rate (%)	0.4	4.7	17.8	33.4	33.52	33.5
	Deaths (per 1000 cases)	0.2027	0.2033	0.1933	0.1847	0.1846	0.1846
25% coverage	Optimal strategy (hospitalizations)	[100 100 0 0]	[100 100 0 0]	[100 100 0 0]	[30 100 0 100]	[30 100 0 100]	[30 100 0 100]
	Illness Attack Rate (%)	0.01	0.42	11.9	33.3	33.5	33.5
	Hospitalizations (per 100 cases)	0.4532	0.4529	0.4531	0.4532	0.4548	0.4550
	Optimal strategy (deaths)	[100 100 0 0]	[100 100 0 0]	[100 100 0 0]	[83 100 0 28]	[30 100 0 100]	[30 100 0 100]
	Illness Attack Rate (%)	0.01	0.42	11.9	33.3	33.5	33.5
	Deaths (per 1000 cases)	0.2089	0.2050	0.1956	0.1843	0.1845	0.1846
