# Supplementary material for: Optimizing Vaccine Allocation at Different Points in Time during an Epidemic
Source: PLoS One. 2010 Nov 11;5(11):e13767. doi: 10.1371/journal.pone.0013767 (PMC2978681; doi:10.1371/journal.pone.0013767)
Supplement: Table S6 — Results for a Less Developed Country, influenza-related mortality and hospitalizations unadjusted, R0 = 1.4. (0.07 MB PDF) [file pone.0013767.s010.pdf]

Table S6: Results for a Less Developed Country, influenza-related mortality and hospitalizations unadjusted, R_0= 1.4.

**Less Developed Country Unadjusted $\mathbf{R_0 = 1.4}$**		Day 1	Day 40	Day 80	Day 90	Day 100	Day 120
2% coverage	Optimal strategy (hospitalizations)	[0 41 0 0]	[0 41 0 0]	[0 41 0 0]	[0 41 0 0]	[0 41 0 0]	[0 41 0 0]
	Illness Attack Rate (%)	24.8	24.8	24.8	25.3	26.1	26.6
	Hospitalizations (per 100 cases)	0.3950	0.3950	0.3953	0.4092	0.4319	0.4442
	Optimal strategy (deaths)	[0 41 0 0]	[0 41 0 0]	[0 41 0 0]	[0 41 0 0]	[0 41 0 0]	[0 41 0 0]
	Illness Attack Rate (%)	24.8	24.8	24.8	25.3	26.1	26.6
	Deaths (per 1000 cases)	0.1073	0.1073	0.1074	0.1093	0.1123	0.1140
15% coverage	Optimal strategy (hospitalizations)	[20 100 0 0]	[20 100 0 0]	[20 100 0 0]	[20 100 0 0]	[6 100 0 74]	[1 100 0 100]
	Illness Attack Rate (%)	2.7	5.61	8.7	16	23.5	26
	Hospitalizations (per 100 cases)	0.3156	0.3181	0.3222	0.3742	0.4040	0.4355
	Optimal strategy (deaths)	[20 100 0 0]	[20 100 0 0]	[1 100 0 100]	[1 100 0 100]	[1 100 0 100]	[1 100 0 100]
	Illness Attack Rate (%)	2.7	5.61	18.5	20.6	24	26
	Deaths (per 1000 cases)	0.0972	0.0990	0.0577	0.0729	0.0965	0.1097
25% coverage	Optimal strategy (hospitalizations)	[40 100 0 0]	[40 100 0 0]	[40 100 0 0]	[40 100 0 0]	[21 100 0 100]	[21 100 0 100]
	Illness Attack Rate (%)	0.02	0.127	0.87	11.37	21.7	25.5
	Hospitalizations (per 100 cases)	0.3501	0.3534	0.3569	0.4008	0.4118	0.4394
	Optimal strategy (deaths)	[40 100 0 0]	[40 100 0 0]	[21 100 0 100]	[21 100 0 100]	[21 100 0 100]	[21 100 0 100]
	Illness Attack Rate (%)	0.02	0.12	5.7	14.3	21.7	25.4
	Deaths (per 1000 cases)	0.1128	0.1113	0.0602	0.0809	0.1009	0.1107
