# Supplementary material for: Optimizing Vaccine Allocation at Different Points in Time during an Epidemic
Source: PLoS One. 2010 Nov 11;5(11):e13767. doi: 10.1371/journal.pone.0013767 (PMC2978681; doi:10.1371/journal.pone.0013767)
Supplement: Table S7 — Results for a Less Developed Country, influenza-related mortality and hospitalizations adjusted, R0 = 1.4. (0.07 MB PDF) [file pone.0013767.s011.pdf]

Table S7: Results for a Less Developed Country, influenza-related mortality and hospitalizations adjusted, R_0= 1.4.

**Less Developed Country Adjusted $\mathbf{R_0 = 1.4}$**		Day 1	Day 40	Day 80	Day 90	Day 100	Day 120
2% coverage	Optimal strategy (hospitalizations)	[0 41 0 0]	[0 41 0 0]	[0 41 0 0]	[0 41 0 0]	[0 41 0 0]	[0 41 0 0]
	Illness Attack Rate (%)	24.8	24.8	24.8	25.3	26.1	26.6
	Hospitalizations (per 100 cases)	2.5465	2.5468	2.5493	2.6628	2.8479	2.9485
	Optimal strategy (deaths)	[0 41 0 0]	[0 41 0 0]	[0 41 0 0]	[0 41 0 0]	[0 41 0 0]	[0 41 0 0]
	Illness Attack Rate (%)	24.8	24.8	24.8	25.3	26.1	26.6
	Deaths (per 1000 cases)	0.5215	0.5216	0.5220	0.5383	0.5651	0.5797
15% coverage	Optimal strategy (hospitalizations)	[20 100 0 0]	[20 100 0 0]	[20 100 0 0]	[20 100 0 0]	[20 100 0 0]	[20 100 0 0]
	Illness Attack Rate (%)	2.7	5.6	8.7	16	22.6	25.8
	Hospitalizations (per 100 cases)	1.8898	1.8914	1.9064	2.3430	2.7618	2.9306
	Optimal strategy (deaths)	[20 100 0 0]	[20 100 0 0]	[20 100 0 0]	[1 100 0 100]	[1 100 0 100]	[1 100 0 100]
	Illness Attack Rate (%)	2.7	5.6	8.7	20.6	24	26
	Deaths (per 1000 cases)	0.4286	0.4333	0.4399	0.3966	0.5078	0.5648
25% coverage	Optimal strategy (hospitalizations)	[40 100 0 0]	[40 100 0 0]	[40 100 0 0]	[40 100 0 0]	[40 100 0 0]	[40 100 0 0]
	Illness Attack Rate (%)	0.02	0.12	0.8	11.3	20.8	25.3
	Hospitalizations (per 100 cases)	2.0352	2.0870	2.1238	2.5373	2.8365	2.9487
	Optimal strategy (deaths)	[40 100 0 0]	[40 100 0 0]	[40 100 0 0]	[21 100 0 100]	[21 100 0 100]	[21 100 0 100]
	Illness Attack Rate (%)	0.02	0.12	0.81	14.3	217	25.5
	Deaths (per 1000 cases)	0.4822	0.4837	0.4872	0.4342	0.5285	0.5703
