# Supplementary material for: Optimizing Vaccine Allocation at Different Points in Time during an Epidemic
Source: PLoS One. 2010 Nov 11;5(11):e13767. doi: 10.1371/journal.pone.0013767 (PMC2978681; doi:10.1371/journal.pone.0013767)
Supplement: Table S8 — Results for a Less Developed Country, influenza-related mortality and hospitalizations unadjusted, R0 = 1.8. (0.07 MB PDF) [file pone.0013767.s012.pdf]

Table S8: Results for a Less Developed Country, influenza-related mortality and hospitalizations unadjusted, R_0= 1.8.

**Less Developed Country Unadjusted $\mathbf{R_0 = 1.8}$**		Day 1	Day 20	Day 40	Day 50	Day 60	Day 70
2% coverage	Optimal strategy (hospitalizations)	[0 41 0 0]	[0 41 0 0]	[0 41 0 0]	[0 41 0 0]	[0 41 0 0]	[0 41 0 0]
	Illness Attack Rate (%)	38.90	38.9	39.3	40	40.4	40.5
	Hospitalizations (per 100 cases)	0.4005	0.4010	0.4154	0.4345	0.4447	0.4479
	Optimal strategy (deaths)	[0 39 0 1]	[0 32 0 5]	[0 0 0 21]	[0 0 0 21]	[0 0 0 21]	[0 0 0 21]
	Illness Attack Rate (%)	38.9	39.1	39.9	40.1	40.4	40.5
	Deaths (per 1000 cases)	0.1173	0.1169	0.1166	0.1196	0.1221	0.1232
15% coverage	Optimal strategy (hospitalizations)	[1 100 0 100]	[1 100 0 100]	[1 100 0 100]	[1 100 0 100]	[1 100 0 100]	[0 100 2 100]
	Illness Attack Rate (%)	31.9	32	33.9	37.1	39.3	40.2
	Hospitalizations (per 100 cases)	0.2664	0.2680	0.3227	0.3934	0.4312	0.4438
	Optimal strategy (deaths)	[1 100 0 100]	[1 100 0 100]	[1 100 0 100]	[0 100 2 100]	[0 100 2 100]	[0 100 2 100]
	Illness Attack Rate (%)	31.9	32	33.9	37.1	39.3	40.2
	Deaths (per 1000 cases)	0.0623	0.0628	0.0780	0.1004	0.1152	0.1210
25% coverage	Optimal strategy (hospitalizations)	[40 100 0 0]	[40 100 0 0]	[21 100 0 100]	[21 100 0 100]	[21 100 0 100]	[3 100 25 100]
	Illness Attack Rate (%)	15.3	16.2	27.7	34.5	38.5	39.9
	Hospitalizations (per 100 cases)	0.3496	0.3521	0.3369	0.4033	0.4346	0.4448
	Optimal strategy (deaths)	[40 100 0 0]	[21 100 0 100]	[21 100 0 100]	[0 100 29 100]	[0 100 29 100]	[0 100 29 100]
	Illness Attack Rate (%)	15.3	22.7	27.7	35.5	38.7	39.9
	Deaths (per 1000 cases)	0.1250	0.0657	0.0827	0.1001	0.1153	0.1211
