# Supplementary material for: Optimizing Vaccine Allocation at Different Points in Time during an Epidemic
Source: PLoS One. 2010 Nov 11;5(11):e13767. doi: 10.1371/journal.pone.0013767 (PMC2978681; doi:10.1371/journal.pone.0013767)
Supplement: Table S9 — Results for a Less Developed Country, influenza-related mortality and hospitalizations adjusted, R0 = 1.8. (0.07 MB PDF) [file pone.0013767.s013.pdf]

Table S9: Results for a Less Developed Country, influenza-related mortality and hospitalizations adjusted, R_0= 1.8.

**Less Developed Country Adjusted $\mathbf{R_0 = 1.8}$**		Day 1	Day 20	Day 40	Day 50	Day 60	Day 70
2% coverage	Optimal strategy (hospitalizations)	[0 41 0 0]	[0 41 0 0]	[0 41 0 0]	[0 41 0 0]	[0 41 0 0]	[0 41 0 0]
	Illness Attack Rate (%)	38.9	38.9	39.3	40	40.4	40.5
	Hospitalizations (per 100 cases)	2.4763	2.4798	2.5984	2.7559	2.8400	2.8664
	Optimal strategy (deaths)	[0 41 0 0]	[0 41 0 0]	[0 41 0 0]	[0 41 0 0]	[0 41 0 0]	[0 41 0 0]
	Illness Attack Rate (%)	38.9	38.9	39.3	40	40.4	40.5
	Deaths (per 1000 cases)	0.5390	0.5395	0.5564	0.5789	0.5909	0.5947
15% coverage	Optimal strategy (hospitalizations)	[20 100 0 0]	[20 100 0 0]	[20 100 0 0]	[8 100 0 60]	[1 100 0 100]	[1 100 0 100]
	Illness Attack Rate (%)	26.8	26.9	31	36.7	39.3	40.2
	Hospitalizations (per 100 cases)	1.8690	1.8812	2.2492	2.6055	2.7926	2.8534
	Optimal strategy (deaths)	[1 100 0 100]	[1 100 0 100]	[1 100 0 100]	[1 100 0 100]	[1 100 0 100]	[0 100 2 100]
	Illness Attack Rate (%)	31.9	32	33.9	37.1	39.3	40.2
	Deaths (per 1000 cases)	0.3266	0.3289	0.4049	0.5075	0.5661	0.5868
25% coverage	Optimal strategy (hospitalizations)	[40 100 0 0]	[40 100 0 0]	[40 100 0 0]	[40 100 0 0]	[40 100 0 0]	[40 100 0 0]
	Illness Attack Rate (%)	15.3	16.2	24.7	34.2	38.5	40
	Hospitalizations (per 100 cases)	1.8795	1.8981	2.3426	2.6613	2.8128	2.8593
	Optimal strategy (deaths)	[40 100 0 0]	[40 100 0 0]	[21 100 0 100]	[21 100 0 100]	[21 100 0 100]	[3 100 29 100]
	Illness Attack Rate (%)	15.3	16.2	27.7	34.5	38.5	39.9
	Deaths (per 1000 cases)	0.4960	0.4990	0.4249	0.5216	0.5710	0.5881
