# Supplementary material for: Optimizing Vaccine Allocation at Different Points in Time during an Epidemic
Source: PLoS One. 2010 Nov 11;5(11):e13767. doi: 10.1371/journal.pone.0013767 (PMC2978681; doi:10.1371/journal.pone.0013767)
Supplement: Text S1 — Supplemental material for ‘Optimizing vaccine allocation at different points in time during an epidemic’. (0.11 MB PDF) [file pone.0013767.s014.pdf]

Supplemental material for “Optimizing vaccine allocation at different points in time during an epidemic”

Laura Matrajt[1] and Ira M. Longini Jr.[2,3]

October 13, 2010

[1]Department of Applied Mathematics, University of Washington, Seattle, WA, USA
[2]Center for Statistics and Quantitative Infectious Diseases, Vaccine and Infectious Disease Division, Fred Hutchinson Cancer Research Center, Seattle, WA, USA
[3]Department of Biostatistics, School of Public Health, University of Washington, Seattle, WA, USA.

Mathematical model

We considered a closed population of size N. We divided the population into two sub-populations of children and adults of size N_1 and N_2, so that $N = N_1 + N_2$. Furthermore, within each sub-population, we divide members into high risk and low risk. Members in each group are either susceptible, infected asymptomatic, infected symptomatic or recovered and immune. In addition, people can be either vaccinated or unvaccinated.

The susceptibles are denoted by S_{lij}, and S_{hij}, infected asymptomatic by A_{lij} and A_{hij}, infected symptomatic by I_{lij} and I_{hij}, recovered asymptomatic by RA_i, and recovered symptomatic by R_{li} and R_{hi} where $i = 1, 2$ denotes the age group (children and adults, respectively), j denotes the vaccination status ($j = 0$ for the unvaccinated and $j = 1$ for the vaccinated), l denotes the low risk group and h denotes the high risk group. A fraction $1-\rho$ of the infected class will not develop symptoms and have their infectiousness reduced by a factor m, with $m \in [0, 1]$. Infected people, both symptomatics and asymptomatics leave the infected classes at a rate γ.

We modeled an imperfect vaccine which has three major effects [1]; a reduction in the susceptibility, a reduction in the infectiousness and a reduction in the pathogenicity (a

reduction in the probability symptoms in infected people). These effects are denoted in the model by $\mathsf{VE_S}$, $\mathsf{VE_I}$ and $\mathsf{VE_P}$, respectively. Based on previous immunogenicity studies (e.g., [2, 3, 4, 5]), we assumed that the vaccine efficacy will reach its full potential 14 days after being administered. During this time, each vaccine efficacy will build up in time in an exponential-like fashion and will remain constant afterward (see figure S1). The function used is

$$f(t) = \exp(t^{0.27}),$$

where the coefficient was arbitrarily chosen for convenience.

Let p be the probability of infection given contact and c_{ij} the contact rate between people in class i and people in class j, where $i = 1$ represents children and $i = 2$ represents adults. We consider symmetry in contacts, so that $c_{ij} = c_{ji}$. Using [6] as a guide, we computed the parameters c_{ij} to obtain the final illness attack rates given in table S3. These attack rates satisfy the condition that the ratio of the proportion of children infected and adults infected approximately matches the estimates for pandemic H1N1 for the United States given in [7].

We obtain the following system of equations for the epidemic system:

Equations for susceptibles

Unvaccinated / Vaccinated

$$\frac{dS_{l10}}{dt} = -\lambda_1 S_{l10} \qquad \frac{dS_{l11}}{dt} = -\lambda_1 \theta S_{l11} \quad (1)$$

$$\frac{dS_{h10}}{dt} = -\lambda_1 S_{h10} \qquad \frac{dS_{h11}}{dt} = -\lambda_1 \theta S_{h11} \quad (2)$$

$$\frac{dS_{l20}}{dt} = -\lambda_2 S_{l20} \qquad \frac{dS_{l21}}{dt} = -\lambda_2 \theta S_{l21} \quad (3)$$

$$\frac{dS_{h20}}{dt} = -\lambda_2 S_{l20} \qquad \frac{dS_{h21}}{dt} = -\lambda_2 \theta S_{l21} \quad (4)$$

Equations for infected asymptomatics

Unvaccinated / Vaccinated

$$\frac{dA_{l10}}{dt} = \lambda_1(1-\rho) S_{l10} - \gamma A_{l10} \qquad \frac{dA_{l11}}{dt} = \lambda_1(1-\rho\psi)\theta S_{l11} - \gamma A_{l11} \quad (5)$$

$$\frac{dA_{h10}}{dt} = \lambda_1(1-\rho) S_{h10} - \gamma A_{h10} \qquad \frac{dA_{h11}}{dt} = \lambda_1(1-\rho\psi)\theta S_{h11} - \gamma A_{h11} \quad (6)$$

$$\frac{dA_{l20}}{dt} = \lambda_2(1-\rho) S_{l20} - \gamma A_{l20} \qquad \frac{dA_{l21}}{dt} = -\lambda_2(1-\rho\psi)\theta S_{l21} - \gamma A_{l21} \quad (7)$$

$$\frac{dA_{h20}}{dt} = \lambda_2(1-\rho) S_{h20} - \gamma A_{h20} \qquad \frac{dA_{h21}}{dt} = \lambda_2(1-\rho\psi)\theta S_{h21} - \gamma A_{h21} \quad (8)$$

Equations for infected symptomatics

Unvaccinated | Vaccinated

$$\frac{dI_{l10}}{dt} = \lambda_1 \rho S_{l10} - \gamma I_{l10} \qquad \frac{dI_{l11}}{dt} = \lambda_1 \rho \psi \theta S_{l11} - \gamma I_{l11} \tag{9}$$
$$\frac{dI_{h10}}{dt} = \lambda_1 \rho S_{h10} - \gamma I_{h10} \qquad \frac{dI_{h11}}{dt} = \lambda_1 \rho \psi \theta S_{h11} - \gamma I_{h11} \tag{10}$$
$$\frac{dI_{l20}}{dt} = \lambda_2 \rho S_{l20} - \gamma I_{l20} \qquad \frac{dI_{l21}}{dt} = \lambda_2 \rho \psi \theta S_{l21} - \gamma I_{l21} \tag{11}$$
$$\frac{dI_{h20}}{dt} = \lambda_2 \rho S_{h20} - \gamma I_{h20} \qquad \frac{dI_{h21}}{dt} = \lambda_2 \rho \psi \theta S_{h21} - \gamma I_{h21} \tag{12}$$

Equations for the recovered

$$\frac{dRA_1}{dt} = \gamma(A_{l10} + A_{l11} + A_{h10} + A_{h11}) \tag{13}$$
$$\frac{dRA_2}{dt} = \gamma(A_{l20} + A_{l21} + A_{h20} + A_{h21}) \tag{14}$$
$$\frac{dRI_{l1}}{dt} = \gamma(I_{l10} + I_{l11}) \tag{15}$$
$$\frac{dRI_{h1}}{dt} = \gamma(I_{h10} + I_{h11}) \tag{16}$$
$$\frac{dRI_{l2}}{dt} = \gamma(I_{l20} + I_{l21}) \tag{17}$$
$$\frac{dRI_{h2}}{dt} = \gamma(I_{h20} + I_{h21}) \tag{18}$$

where $\theta = 1 - \mathsf{VE_S}$ $\phi = 1 - \mathsf{VE_I}$ and $\psi = 1 - \mathsf{VE_P}$. The forces of infection are given by

$$\lambda_1 = \frac{pc_{11}}{N_1}\Big(m(A_{l10} + A_{h10}) + m\phi(A_{l11} + A_{h11}) + I_{l10} + I_{h10} + \phi(I_{l11} + I_{h11})\Big) + \frac{pc_{12}}{N_2}\Big(m(A_{l20} + A_{h20}) + m\phi(A_{l21} + A_{h21}) + I_{l20} + I_{h20} + \phi(I_{l21} + I_{h21})\Big),$$

and

$$\lambda_2 = \frac{pc_{21}}{N_1}\Big(m(A_{l10} + A_{h10}) + m\phi(A_{l11} + A_{h11}) + I_{l10} + I_{h10} + \phi(I_{l11} + I_{h11})\Big) + \frac{pc_{22}}{N_2}\Big(m(A_{l20} + A_{h20}) + m\phi(A_{l21} + A_{h21}) + I_{l20} + I_{h20} + \phi(I_{l21} + I_{h21})\Big).$$

The parameter values used are given in table S2.

Given the variability of the estimates for the basic reproduction number for pandemic influenza H1N1 [8, 9, 10], we considered three different basic reproduction numbers $R_0 = 1.4$, $R_0 = 1.6$ and $R_0 = 1.8$. The basic reproduction numbers were computed following the approach given in [11] and [12, 13]. We varied the value of p, the probability of transmission, to obtain the desired value of R_0.

Implementation of vaccination

We considered six different possibilities for starting vaccination: 1. the first day of the epidemic, 2. very early on in the epidemic, 3. before the exponential phase of the epidemic, 4. during the exponential phase of the epidemic, 5. just before the peak of the epidemic, and 6. just after the peak of the epidemic. Since for each R_0 the speed of the epidemic is different, we manually picked, for each R_0, six starting times corresponding to each of the six possibilities. Table S1 summarizes these times.

Numerical implementation was done as follows. If we were to vaccinate fractions f_{l1} and f_{h1} of children low and high risk and fractions f_{l2} and f_{h2} of adults low and high risk starting on day τ, then we would run the system up to day τ, and vaccinate each group by removing the corresponding fraction from each of the susceptible classes and adding it to the respective vaccinated class. Hence we have, corresponding to day τ, a time step t with the following conditions:

$$
\begin{aligned}
S_{l10}(t+1) &= (1 - f_{l1})S_{l10}(t) & S_{l11}(t+1) &= f_{l1}S_{l10}(t) \\
S_{h10}(t+1) &= (1 - f_{h1})S_{h10}(t) & S_{h11}(t+1) &= f_{h1}S_{h10}(t) \\
S_{l20}(t+1) &= (1 - f_{l2})S_{l20}(t) & S_{l21}(t+1) &= f_{l2}S_{l20}(t) \\
S_{h20}(t+1) &= (1 - f_{h2})S_{h20}(t) & S_{h21}(t+1) &= f_{h2}S_{h20}(t).
\end{aligned}
$$

Optimization

We define a vaccination control vector $\boldsymbol{f} = (f_{l1}, f_{h1}, f_{l2}, f_{h2})$ where f_{l1} and f_{h1} are the fractions of vaccinated children at low and high risk, respectively, and f_{l2} and f_{h2} are the fractions of vaccinated adults at low and high risk, respectively.

We define an objective function $g(\boldsymbol{f}) = g(f_{l1}, f_{h1}, f_{l2}, f_{h2})$ as follows:

$$
\begin{aligned}
g(\boldsymbol{f}) = g(f_{l1}, f_{h1}, f_{l2}, f_{h2}) = a_{1l} \cdot RI_{l1} + a_{1h} \cdot RI_{h1} + \\
a_{2l} \cdot RI_{l1} + a_{2h} \cdot RI_{h2}
\end{aligned} \tag{19}
$$

where a_{il} is the fraction of deaths or hospitalizations in the infected symptomatic low-risk subgroup, and a_{ih} is the fraction of deaths or hospitalizations in the infected symptomatic high-risk subgroup ($i = 1, 2$). This function is the expected number of deaths or hospitalizations in each subgroup (i.e., children, low and high risk; adults, low and high risk) and is computed as a weighted average, with weights defined in table S2. Then, we have the optimization problem

$$\min_{\boldsymbol{f}} g(f_{l1}^*, f_{h1}^*, f_{l2}^*, f_{h2}^*) = g(\boldsymbol{f^*}), \tag{20}$$

$$\tag{21}$$

subject to the constraints

$$0 \le f_{l1}, f_{h1}, f_{l2}, f_{h2} \le 1,$$
$$f_{l1}(1-\delta_1)N_1 + f_{h1}\delta_1 N_1 + f_{l2}(1-\delta_2)N_2 + f_{h2}\delta_2 N_2 = T,$$

where T is the total number of vaccine doses available, and δ_1 and δ_2 are the fractions of children and adults at high risk, respectively. Using a line search algorithm found in the optimization package in MATLAB, we were able to find the optimal vaccine distribution under each scenario considered.

Results

The tables S4 and S5 summarize the results for the optimal strategy in a DC for a basic reproduction number of 1.4 and 1.8 respectively. Similarly, the tables S6-S9 summarize the results for a LDC, both with influenza-related mortality and hospitalizations unadjusted (tables S6 and S8) and adjusted (tables S7 and S9).

Sensitivity analysis

We performed sensitivity analysis for the basic reproduction numbers (figure S2). As the basic reproduction number increases, the optimal strategy shifts in general from low-risk children to high-risk adults. We also performed sensitivity analysis for the parameters that account for the excess of influenza-related mortality and hospitalizations in a LDC. In order to do this, we repeated the analysis for $R_0 = 1.6$ where these multipliers were taken to be half of the ones considered in table S2 (increase in the influenza-related mortality by a

factor of four instead of eight in children and 1.5 instead of three in adults). Figures S3 and S4 show the percentage of the total number of doses used in each group in a less developed country where the multipliers were not adjusted, (left panel), adjusted as described above (center panel) and adjusted as described in the main text (right panel). Figure S3 shows the percentage of doses used when there is enough vaccine to cover 15% of the population and the optimizer was set to minimize hospitalizations, whereas figure S4, there is enough vaccine to cover 25% of the population and the optimizer was set to minimize mortality. In both cases, increasing these multipliers, that is, increasing the excess of influenza-related mortality or hospitalizations tends to favor the high-transmission group, the children at low risk.

References

[1] Halloran ME, Struchiner CJ, Longini IM (1997) Study designs for evaluating different efficacy and effectiveness aspects of vaccines. American Journal of Epidemiology 146: 789–803.

[2] National Institute of Allergy and Infectious Diseases, National Institutes of Health (2009). Early Results: In Children, 2009 H1N1 Influenza Vaccine Works Like Seasonal Flu Vaccine. `http://www3.niaid.nih.gov/news/newsreleases/2009/H1N1PedTrial.htm`.

[3] National Institute of Allergy and Infectious Diseases, National Institutes of Health (2009). Updated Results: In Youngest Children, a Second Dose of 2009 H1N1 Influenza Vaccine Elicits Robust Immune Response. . `UpdatedResults:InYoungestChildren, aSecondDoseof2009H1N1InfluenzaVaccineElicitsRobustImmuneResponse.`.

[4] Plennevaux E, Sheldon E, Blatter M, Reeves-Hoche MK, Denis M (2010) Immune response after a single vaccination against 2009 influenza A H1N1 in USA: a preliminary report of two randomised controlled phase 2 trials. Lancet 375: 41–48.

[5] Nolan T, McVernon J, Skeljo M, Richmond P, Wadia U, et al. (2010) Immunogenicity of a monovalent 2009 influenza A(H1N1) vaccine in infants and children: a randomized trial. JAMA 303: 37–46.

[6] Hill AN, Longini IM (2003) The critical vaccination fraction for heterogeneous epidemic models. Mathematical Biosciences 181: 85-106.

[7] Centers for Disease Control and Prevention (2010). CDC Estimates of 2009 H1N1 Influenza Cases, Hospitalizations and Deaths in the United States, April – December 12, 2009. http://www.cdc.gov/h1n1flu/estimates/April_December_12.htm?s_cid=cs_000. last accesed Feb. 17th 2010.

[8] Yang Y, Sugimoto JD, Halloran ME, Basta NE, Chao DL, et al. (2009) The transmissibility and control of pandemic influenza A (H1N1) virus. Science 326: 729-33.

[9] Fraser C, Donnelly CA, Cauchemez S, Hanage WP, Van Kerkhove MD, et al. (2009) Pandemic potential of a strain of influenza A (H1N1): early findings. Science 324: 1557–1561.

[10] Balcan D, Hu H, Goncalves B, Bajardi P, Poletto C, et al. (2009) Seasonal transmission potential and activity peaks of the new influenza A(H1N1): a Monte Carlo likelihood analysis based on human mobility. BMC Med 7: 45.

[11] Diekmann O, Heesterbeek J, Metz J (1990) On the definition and computation of the basic reproduction ratio R0 in models for infectious-diseases in heterogeneous populations. Journal of Mathematical Biology 28: 365-382.

[12] van den Driessche P, Watmough J (2002) Reproduction numbers and sub-threshold endemic equilibria for compartmental models of disease transmission. Mathematical Biosciences 180: 29-48.

[13] Brauer F, van den Driessche P, Wu J, editors (2008) Mathematical Epidemiology, volume 1945 of *Lecture Notes in Mathematics*. Springer.
